# Endogenous piRNA-guided slicing triggers responder and trailer piRNA production from viral RNA in *Aedes aegypti* mosquitoes

**DOI:** 10.1093/nar/gkab640

**Published:** 2021-07-31

**Authors:** Joep Joosten, Gijs J Overheul, Ronald P Van Rij, Pascal Miesen

**Affiliations:** Department of Medical Microbiology, Radboud Institute for Molecular Life Sciences, Radboud University Medical Center, Nijmegen, P.O. Box 9101, 6500 HB, The Netherlands; Department of Medical Microbiology, Radboud Institute for Molecular Life Sciences, Radboud University Medical Center, Nijmegen, P.O. Box 9101, 6500 HB, The Netherlands; Department of Medical Microbiology, Radboud Institute for Molecular Life Sciences, Radboud University Medical Center, Nijmegen, P.O. Box 9101, 6500 HB, The Netherlands; Department of Medical Microbiology, Radboud Institute for Molecular Life Sciences, Radboud University Medical Center, Nijmegen, P.O. Box 9101, 6500 HB, The Netherlands

## Abstract

In the germline of animals, PIWI interacting (pi)RNAs protect the genome against the detrimental effects of transposon mobilization. In *Drosophila*, piRNA-mediated cleavage of transposon RNA triggers the production of responder piRNAs via ping-pong amplification. Responder piRNA 3′ end formation by the nuclease Zucchini is coupled to the production of downstream trailer piRNAs, expanding the repertoire of transposon piRNA sequences. In *Aedes aegypti* mosquitoes, piRNAs are generated from viral RNA, yet, it is unknown how viral piRNA 3′ ends are formed and whether viral RNA cleavage gives rise to trailer piRNA production. Here we report that in *Ae. aegypti*, virus- and transposon-derived piRNAs have sharp 3′ ends, and are biased for downstream uridine residues, features reminiscent of Zucchini cleavage of precursor piRNAs in *Drosophila*. We designed a reporter system to study viral piRNA 3′ end formation and found that targeting viral RNA by abundant endogenous piRNAs triggers the production of responder and trailer piRNAs. Using this reporter, we identified the *Ae. aegypti* orthologs of Zucchini and Nibbler, two nucleases involved in piRNA 3′ end formation. Our results furthermore suggest that autonomous piRNA production from viral RNA can be triggered and expanded by an initial cleavage event guided by genome-encoded piRNAs.

## INTRODUCTION

Blood-feeding mosquitoes of the *Aedes* genus are responsible for the transmission of arthropod-borne (arbo)viruses that cause severe diseases, such as dengue, Zika, chikungunya and yellow fever. For efficient transmission to occur, arboviruses have to actively replicate in several mosquito tissues to eventually infect the salivary gland (1). Therefore, suppression of virus replication by the mosquito antiviral immune response strongly affects the efficiency of arboviral spread. The cornerstone of antiviral immunity in insects is the small interfering (si)RNA pathway, in which viral double stranded (ds)RNA is cleaved by Dicer-2 into siRNAs (2). These siRNAs provide sequence specificity to the endonuclease Argonaute 2 to direct the cleavage of single stranded viral transcripts. Intriguingly, in *Aedes* mosquitoes, viral RNA is also processed by a somatically active PIWI interacting (pi)RNA pathway, suggesting that two independent small RNA pathways act in parallel to combat viral infections (3).

The piRNA biogenesis machinery has been thoroughly characterized in the model organism *Drosophila melanogaster*, where a gonad-restricted piRNA pathway defends the germline genome from parasitic genetic elements called transposons (4,5). piRNA biogenesis is initiated by the cleavage of genome-encoded piRNA precursors, which are rich in transposon remnants. Processing of these precursor transcripts into pre-piRNAs is mediated either by a piRNA-guided PIWI protein or the endonuclease Zucchini (Zuc), which operates independently of small RNAs (6–8). Pre-piRNAs are loaded into the PIWI proteins Aubergine (Aub) and Piwi, where their 3′ ends may be further trimmed by the exonuclease Nibbler (Nbr), followed by Hen1-mediated 2′-*O*-methylation to generate mature piRNAs (9–13). Whereas Piwi translocates to the nucleus to silence transposons at the transcriptional level (14,15), Aub remains in the cytoplasm where it cleaves (slices) transposon mRNA with sequence complementarity to its associated piRNA (16,17). The resulting cleavage fragments are loaded into the PIWI protein Argonaute 3 (Ago3) and matured into responder piRNAs by Zuc cleavage and/or Nbr-mediated trimming and subsequent 2′-*O*-methylation by Hen1 (11,13). In turn, these responder piRNAs direct Ago3-mediated cleavage of piRNA precursors, triggering the production of new initiator piRNAs, completing the so-called ping-pong loop (16–19).

In *Drosophila*, Zuc-mediated generation of piRNA 3′ ends releases a downstream cleavage product that is preferentially loaded into Piwi, thereby generating a new pre-piRNA. This mechanism results in phased processing of piRNA precursor transcripts into a string of piRNAs named trailer piRNAs (7,8). Thus, the ping-pong loop amplifies those piRNAs that initially recognized active transposons, while phased trailer piRNA production expands the piRNA sequence repertoire for more efficient repression of transposons. Historically, piRNAs derived from cluster transcripts and transposon mRNAs were termed primary and secondary piRNAs, respectively. Hereafter, we use the terms initiator and responder for ping-pong amplified piRNAs and trailer for piRNAs produced through phased biogenesis, as proposed in (4).

The *Aedes aegypti* piRNA pathway is also involved in transposon control and has recently been shown to generate trailer piRNAs (6). However, the pathway differs from that in *Drosophila* in four important ways: (i) the pathway is active in somatic tissues as well as germline tissues (20,21), (ii) the PIWI gene family has expanded to seven members compared to three in *Drosophila* (20,22,23), of which the PIWI proteins Piwi5 and Ago3 engage in ping-pong amplification of piRNAs (24,25), (iii) the *Aedes* piRNA pathway processes non-canonical substrates such as viral RNA (24,26,27), and *iv)* mosquito piRNA clusters contain large numbers of endogenous viral elements (EVEs), sequences of non-retroviral RNA viruses inserted in host genomes (28–30). As a consequence, EVEs give rise to abundant piRNAs (28,31–33) and mediate antiviral defense (34,35). It has been shown that EVE-derived piRNAs can trigger the production of piRNAs from viral RNAs (34,35). Bases on this observation, we propose that, through trailer piRNA production, a single endogenous initiator piRNA can induce the production of an expanded pool of viral piRNAs, thereby enforcing autonomous piRNA production from viral RNA.

Hitherto, piRNA 3′ end formation and generation of trailer piRNAs have not been studied mechanistically in mosquitoes. Here, we demonstrate that *Ae. aegypti* piRNAs, both of transposon and viral origin, display sequence features indicative of a Zucchini-like biogenesis mechanism. We establish a viral piRNA reporter system to show that AAEL011385 and AAEL005527, the *Ae. aegypti* orthologs of *Drosophila* Zuc and Nibbler, respectively, cooperatively determine piRNA 3′ ends. Furthermore, we demonstrate that cleavage guided by a genome-encoded initiator piRNA triggers the production of trailer piRNAs from the viral genome. We propose that piRNA biogenesis triggered from endogenous sequences, in particular EVEs, may equip *Aedes* mosquitoes with a heritable immune response that, through phasing, is able to adapt to newly encountered and continuously mutating viruses.

## MATERIALS AND METHODS

### Cell culture, dsRNA transfection and infection of Aag2 and U4.4 cells

*Ae. aegypti* Aag2 and *Ae. albopictus* U4.4 cells were maintained in supplemented Leibovitz's L-15 medium (Invitrogen) at 25°C. For knockdown experiments, dsRNA was transfected using X-tremeGENE HP DNA Transfection Reagent (Roche) according to the manufacturer's instructions. Where indicated, cells were infected with Sindbis virus (SINV) at a multiplicity of infection (MOI) of 0.1. For further details, see Supplemental Information.

### Generation of reporter viruses

Target sites for *gypsy*- and EVE-initiator piRNAs and trailer cassettes were introduced into an infectious cDNA clone of Sindbis virus downstream of a duplicated subgenomic promoter. Site-directed mutagenesis was used to introduce target site mutations. Subsequently, viruses were grown as described previously (27). For details, see Supplemental Information.

### RNA isolation, RT-qPCR and small RNA northern blotting

Total RNA was isolated using RNA-SOLV reagent (Omega Bio-tek). For RT-qPCR analyses, RNA was DNaseI treated, reverse transcribed, and PCR amplified in the presence of SYBR green. For small RNA northern blotting, RNA was resolved by denaturing urea polyacrylamide gel electrophoresis and transferred to nylon membranes. See Supplemental Information for experimental details and oligonucleotide sequences.

### Generation of small RNA deep sequencing libraries and bioinformatic analyses

For the analyses of small RNAs, deep sequencing libraries were generated using the NEBNext Small RNA Library Prep Set for Illumina (E7560, New England Biolabs) and sequenced on an Illumina Hiseq4000. Sequence data have been deposited in the NCBI sequence read archive under SRA accession SRP272125. Sequencing data were analyzed in Galaxy (36). Reads were mapped to Sindbis virus genomes, transposon sequences, *Ae. aegypti* transcripts, the Phasi Charoen like virus genome and pre-miRNA sequences using Bowtie (37). Further details are provided in the Supplemental Information.

### Immunofluorescence analyses of Zuc localization

Aag2 cells were transfected with a plasmid expressing 3 × flag tagged Zuc using X-tremeGENE HP DNA Transfection Reagent (Roche), and fixed 48 hours after transfection. Cells were incubated with a mouse anti-flag antibody (Sigma, F1804, RRID: AB_262044), followed by goat anti-mouse IgG Alexa fluor 568 (Invitrogen, A-11004, RRID: AB_2534072). Mitochondria were stained using Mitoview Green (Biotium). For further information, see Supplemental Information.

### Immunoprecipitation and western blot

A 3 × flag tagged Zuc expression plasmid was transfected into Aag2 cells using X-tremeGENE HP DNA Transfection Reagent (Roche). Cells were lysed and lysates incubated with M2-Flag beads (Sigma) to immunoprecipitate 3 × flag tagged Zuc and interacting proteins. For western blot analyses, samples were resolved on polyacrylamide gels, blotted to nitrocellulose membranes and stained with the following antibodies generated in our laboratory (25,38): rabbit-anti-Ago3, -Piwi4, -Piwi5 and -Piwi6 (all at 1:500), and mouse anti-flag (1:1000, Sigma, F1804, RRID: AB_262044). Subsequently, goat-anti-rabbit-IRdye800 [Li-cor; 926-32211, RRID: AB_621843] and goat-anti-mouse-IRdye680 [926-68070, RRID: AB_10956588] were used for visualization. Small RNAs were isolated from PIWI protein immunoprecipitates as described in (25). For experimental details, see Supplemental Information.

### Statistical analyses

Unless indicated otherwise, unpaired two tailed t-tests with Holm-Sidak correction for multiple comparisons were used for statistical analyses (* *P* < 0.05; ** *P* < 0.005; *** *P* < 0.0005) using Prism 8 (GraphPad Software). For statistical analysis of sharpness scores in Figure 4F and G, see Supplemental Information.

## RESULTS AND DISCUSSION

### *Aedes aegypti* piRNAs have sharp 3′ ends

**Figure 1.**
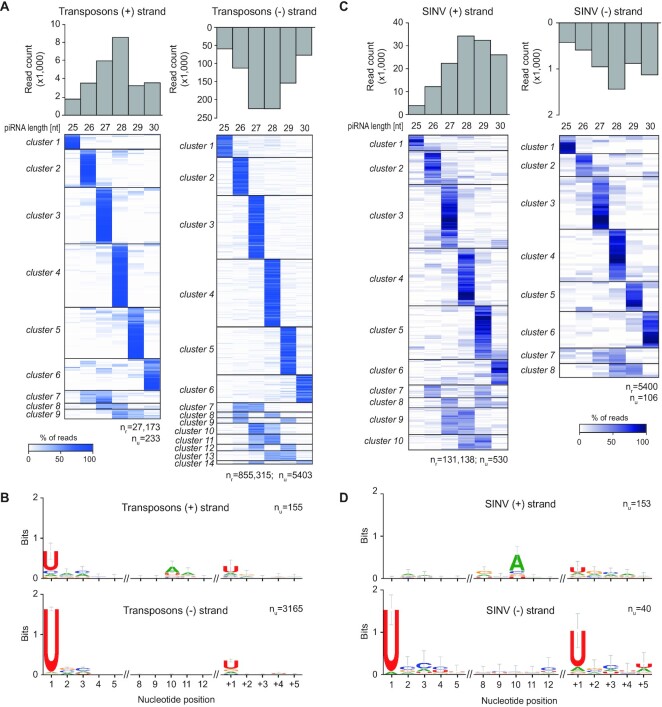
*Aedes aegypti* piRNAs have sharp 3′ ends. (**A**) Analysis of individual piRNA lengths. The bar graphs show the size distribution of transposon-derived sense ((+), left) and antisense ((-), right) piRNAs from the combined three dsLuc small RNA libraries published in (24). The heat maps show the relative size distribution of transposon-derived piRNAs that share the same 5′ end (one 5′ end is one line in the heat map). Shades of blue indicate the percentage of reads contributing to the indicated piRNA length, white represents absence of reads of the specific size. The number of reads (n_r_) and the number of unique piRNAs 5′ ends (n_u_) that underlie the heat map are indicated. A minimum of 20 reads per unique piRNA position was required to be included in the analysis. (**B**) Nucleotide biases at the indicated positions of transposon-derived piRNAs and the sequence at the genomic region directly downstream (+1 until + 5) of the piRNA 3′ ends. Only piRNAs from (A) that had a dominant piRNA length (at least 75% of reads were of the same size) were considered in this analysis and only unique piRNA sequences were analyzed, irrespective of read count. *n*_u_ indicates the number of sequences underlying the sequence logo. (**C** and **D**) The same analysis as for A and B was applied to SINV-derived piRNAs.

In *Drosophila*, piRNA 3′ end formation is largely dependent on the cleavage of pre-piRNAs by the endonuclease Zucchini (Zuc). Zuc uses a sequence motif to preferentially cleave upstream of uridine residues *in vivo* (39–41), hence, piRNAs generated by Zuc generally have sharp 3′ ends and the nucleotide directly downstream of the 3′ end is biased towards uridine (+1U bias) (7,8). We examined whether these characteristics were present in our previously generated small RNA deep sequencing libraries from *Ae. aegypti* Aag2 cells infected with Sindbis virus (SINV) (24). We first analyzed transposon-derived piRNAs and found that piRNAs that shared the same 5′ end generally had the same length (Figure 1A). Specifically, for almost 60% of piRNAs the dominant length made up more than 75% of sequenced reads. We selected these piRNAs and inspected the identity of the nucleotides downstream of that most abundant piRNA isoform. We found that the nucleotide position directly following the 3′ end of the piRNA was biased for uridine (Figure 1B), strongly indicating that these piRNAs were generated by a mechanism resembling Zuc cleavage in *Drosophila*.

We next analyzed the characteristics of 3′ ends of viral (v)piRNAs derived from the SINV genome. SINV is a positive-strand RNA virus of the *Togaviridae* family. During its replication cycle, genomic sense (+) strand RNA serves as a template for the production of antigenomic antisense (-) strand RNA, which in turn provides a template for production of genomic and subgenomic RNA species (42). Strikingly, sharp 3′ ends were clearly visible for vpiRNAs, irrespective of the strand from which the piRNAs were produced (Figure 1C). In addition, a clear + 1U bias was observed, especially for antisense strand derived piRNAs (Figure 1D). These findings suggest that 3′ ends of both Ago3-associated (+) strand-derived vpiRNAs and Piwi5-bound (-) strand-derived vpiRNAs (24,25), are generated, at least in part, by Zuc-like cleavage events. Interestingly, we also observed sharp 3′ ends and + 1U biases for vpiRNAs generated from Phasi Charoen-like virus (Supplementary Figure S1A-B), a negative-strand RNA virus from the *Phenuiviridae* family that persistently infects Aag2 cells (43). These findings indicate that a Zuc-like biogenesis mechanism contributes to 3′ end formation of piRNAs derived from transposons, as well as RNA viruses from diverse families.

### Genome-encoded piRNAs trigger production of virus-derived responder piRNAs

**Figure 2.**
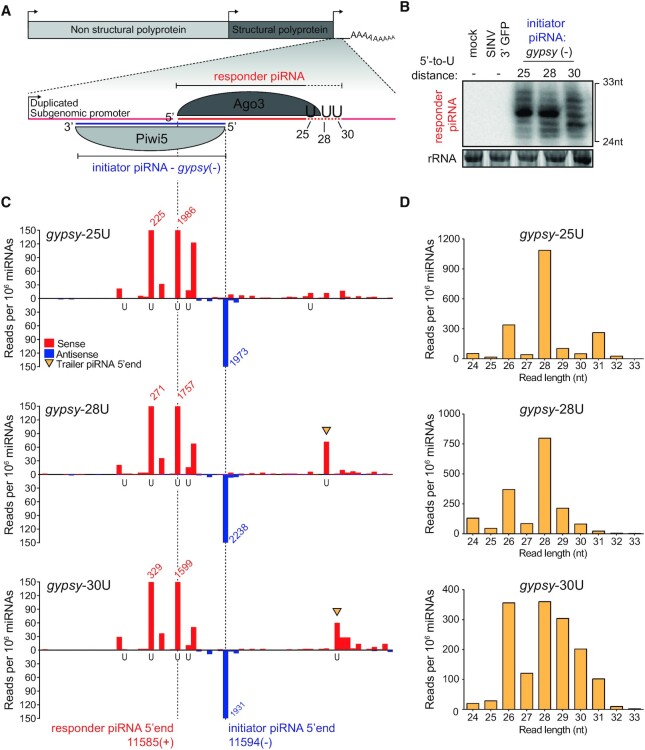
An endogenous piRNA is able to trigger production of virus-derived responder piRNAs. (**A**) Schematic representation of recombinant Sindbis reporter viruses. The enlarged view depicts the reporter locus expressed under the control of a duplicated subgenomic promoter. This non-coding RNA harbors a target site for a Piwi5-associated initiator piRNA derived from the *Ty3*-*gypsy* retrotransposon (*gypsy* - indicated in blue). Slicing of this target triggers the production of responder piRNAs (indicated in red) that are loaded into Ago3. The positions of downstream uridine residues in the various viruses used in later experiments are shown. (**B**) Northern blot analyses of responder piRNAs in Aag2 cells infected with the indicated reporter viruses. Numbers indicate the distance between the 5′ end of the responder piRNA and the first downstream uridine. SINV 3′ GFP is a virus without an initiator piRNA target site and serves as a negative control, as do mock infected cells. The positions marking 24 and 33 nt are inferred from an EtBr-stained small RNA size marker. EtBr-stained rRNA serves as loading control. The northern blot is representative of four independent experiments. (**C**) Visualization of 5′ ends of sense (red) and antisense (blue) piRNAs (24–33 nt) mapping to the reporter locus of the indicated viruses. Dashed lines indicate initiator and responder piRNA 5′ ends and the positions of uridine residues on the sense strand of the various viruses are shown below the x-axis. The red and blue numbers show piRNA counts (in reads per 10^6^ miRNAs) that exceed the range of the y-axis; yellow arrowheads indicate the 5′ ends of trailer piRNAs. For each virus infection, a single sequencing library was analyzed (same for (D)). (**D**) Size distribution of responder piRNAs (SINV genome position 11585 (+)) produced from the various *gypsy*-viruses, as determined by small RNA deep sequencing. Read counts were normalized to the number of miRNAs in each library.

To study vpiRNA 3′ end formation, we designed a SINV-based reporter system which contained a duplicated subgenomic promoter driving the expression of a non-coding RNA sequence that harbors a target site for an abundant initiator piRNA (referred to as reporter cassette, Figure 2A and Supplementary Figure S2A). These Piwi5-associated initiator piRNAs (Supplementary Figure S2B-C) either derived from the Ty3/*gypsy* LTR retrotransposon *gypsy73* (Figure 2A) or from an EVE sequence of flaviviral origin (Supplementary Figure S2A; see also Supplemental text). From here on, we will refer to these piRNAs as *gypsy* and EVE initiator piRNAs. Initiator piRNA-guided recognition of the artificial target site in the reporter virus is expected to trigger slicing by Piwi5 and subsequent processing of the resulting cleavage fragment into an Ago3-associated responder piRNA through ping-pong amplification. Indeed, virus-derived responder piRNAs were abundantly produced in Aag2 cells infected with the reporter viruses containing the artificial piRNA target sites but not in uninfected cells and cells infected with a control virus expressing GFP from the duplicated subgenomic promoter (SINV 3′ GFP) (Figure 2B; Supplementary Figure S2D). These results indicate that endogenous piRNAs can instruct the cleavage of exogenous viral RNA and induce the production of responder piRNAs during acute infection.

Our previous results indicated that both transposon- and SINV-derived piRNAs have a strong bias towards a uridine residue directly downstream of their 3′ ends (Figure 1B, D). To study the importance of the + 1U position for viral responder piRNA production, we introduced uridine residues at specified distances from the putative Piwi5 slice site in the viral reporter (Figure 2A; viruses were named *gypsy*-25U, 28U, and 30U according to the distance of responder piRNA 5′ end to the + 1U). Responder piRNAs were readily detected by high resolution northern blotting for all reporter viruses (Figure 2B), yet the size of the responder piRNA did not reflect the distance between the Piwi5 cleavage site and the downstream uridine residue. While no clear differences in responder piRNA size distribution were observed between the *gypsy*-25U and *gypsy*-28U viruses, increasing the 5′ end-to-U distance to 30 nt (*gypsy*-30U) resulted in a more diffuse pattern of responder piRNA lengths (Figure 2B). These data suggest that downstream uridines are not the only determinant for 3′ end formation of the reporter-derived responder piRNAs or that additional exonucleolytic trimming of pre-piRNA 3′ ends masked a putative endonucleolytic cleavage event directly upstream of the uridine residues. To discriminate between these two possibilities, we analyzed small RNA sequences from Aag2 cells infected with the various reporter viruses. Mapping of piRNA 5′ ends to the genomes of *gypsy*-targeted viruses revealed that virtually no antisense piRNAs other than the initiator piRNAs map to the reporter sequence (Figure 2C). These initiator piRNAs triggered the production of highly abundant sense responder piRNAs with a characteristic 10 nt overlap of piRNA 5′ ends, indicative of production by ping-pong amplification (Figure 2C). Responder piRNA size distribution for *gypsy*-targeted viruses generally recapitulated the results from the northern blot analysis, with a broader size distribution for the *gypsy*-30U virus (Figure 2D).

In viruses with a distance of responder piRNA 5′ ends to + 1U ≥ 28 nt, we detected putative trailer piRNAs downstream of the responder piRNA (indicated with yellow arrowheads in Figure 2C). Strikingly, the 5′ end of these trailer piRNAs was sharply defined by the position of the downstream uridine. Similarly, we observed U-directed trailer piRNA production for the EVE-triggered viruses (Supplementary Figure S2E, Supplemental text). These data suggest that downstream uridines may instruct the positioning of endonucleolytic cleavage, thus coupling responder piRNA 3′ end formation to trailer piRNA 5′ end formation, as previously described in *Drosophila* (7,8). The heterogeneous responder piRNA size likely results from subsequent exonucleolytic trimming.

Intriguingly, only minor U-directed trailer piRNA production was observed in cells infected with the *gypsy*-25U virus. We speculated that the uridine at position 25 may be covered by the Ago3 protein, rendering it inaccessible for endonucleolytic cleavage (Figure 2A). In line with this hypothesis, we observed that only very few responder piRNAs < 26 nt are produced from any *gypsy* triggered reporter virus (Figure 2D). Furthermore, small RNA sequencing data from Ago3 immunoprecipitates (IP) indicated a clear preference of Ago3, Piwi5 and Piwi6 to bind piRNAs in the size range of 26–30 nt (Supplementary Figure S3A, C, D), whereas the Piwi4 IP library was dominated by tapiR1, a highly abundant piRNA that is 30 nt in size (38) (Supplementary Figure S3B). These data strongly support the notion that the lack of trailer piRNAs in the *gypsy*-25U reporter virus is explained by inaccessibility of the introduced U residue due to steric hindrance by the associated PIWI protein. Altogether, these data indicate that our viral reporter system faithfully recapitulates various aspects of piRNA 3′ end formation, serving as an amenable tool to study responder and trailer piRNA biogenesis in *Aedes aegypti*.

### Responder piRNAs are produced through ping-pong mediated slicing

**Figure 3.**
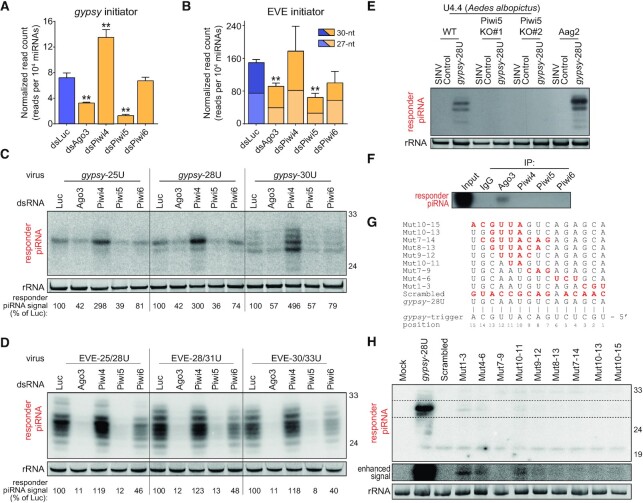
Responder vpiRNAs are produced through ping-pong mediated slicing. (**A**, **B**) Levels of the *gypsy*- (A) and EVE-derived (B) initiator piRNAs in previously published small RNA sequencing libraries generated from Aag2 cells in which indicated PIWI proteins were depleted (24). The flavivirus EVE-derived piRNA exists as 27-nt and 30-nt isoforms; the error bars and statistical analyses are based on combined counts of the two isoforms. Asterisks denote statistical significance as determined by unpaired two tailed t-tests with Holm-Sidak correction (** *P* < 0.005). Bars and whiskers represent the mean and SD of three independent libraries, respectively. (**C**, **D**) Northern blot analysis of viral responder piRNAs in Aag2 cells in which indicated PIWI proteins were depleted. Cells are infected with indicated *gypsy* (C) and EVE (D) targeted viruses. EtBr-stained rRNA serves as loading control. Numbers indicate responder piRNA signals, normalized to the loading control, as a percentage of the responder piRNA signal in dsLuc treated cell infected with the same virus. For both (C) and (D), *n* = 1 biological replicate. (**E**) Northern blot analysis of responder piRNAs in wildtype (WT) and two independent Piwi5 knockout (KO) *Ae. albopictus* U4.4 cell lines, and in *Ae. aegypti* Aag2 cells infected with the *gypsy*-28U virus or a virus that does not express the reporter locus from the second subgenomic promoter (SINV Control). EtBr-stained rRNA serves as a loading control. Northern blot is a representative of two independent biological replicates. (**F**) Northern blot analysis of the viral responder piRNA in PIWI protein immunoprecipitates (IP) from Aag2 cells infected with the *gypsy-*U interval virus (*n* = 1 biological replicate). The *gypsy*-U interval virus, described in detail in Figure 6, contains the same initiator piRNA target site and responder sequence as the *gypsy-*28U virus. As a control, non-specific rabbit IgG was used for IP. (**G**) Overview of target site mutations for the various viruses shown in (H). Red bold font indicates residues that are mismatched with the gypsy initiator piRNA and the numbers denote positions relative to the *gypsy* initiator piRNA 5′ end. (**H**) Northern blot analysis of responder piRNAs in Aag2 cells infected with indicated (mutant) viruses (*n* = 1 biological replicate). The dashed box denotes an area for which the contrast was adjusted to enhance weak responder piRNA signals (enhanced signal – middle panel). The ‘minimal responder’ probe used in this experiment hybridizes to the last 18 nt of the 3′ end of responder piRNAs, which are identical for all viruses (see also Supplementary Figure S4G). EtBr stained rRNA serves as loading control (bottom panel).

We previously identified Ago3 and Piwi5 as the core components of the ping-pong amplification loop in *Ae. aegypti* (24,25). We therefore set out to validate that these PIWI proteins are responsible for the generation of the responder piRNAs from our reporter viruses. First, we determined the levels of *gypsy* and EVE initiator piRNAs in previously published small RNA deep sequencing libraries generated from Aag2 cells in which somatic PIWI proteins (Ago3 and Piwi4-6) were depleted (24). In accordance with ping-pong dependent production, the level of the *gypsy* initiator piRNA was significantly reduced upon knockdown of Ago3 and Piwi5 (2.2- and 5.5-fold, respectively; Figure 3A). Similarly, EVE-derived initiator piRNA levels were significantly reduced upon knockdown of the ping-pong partners Ago3 and Piwi5 (1.6-fold and 2.3-fold, respectively, Figure 3B). Unexpectedly, while Piwi4 depletion had previously been reported to cause a decline of piRNAs from a large proportion of transposons (24,34), knockdown of Piwi4 resulted in an almost twofold increase in *gypsy* initiator piRNAs (Figure 3A). Moreover, Piwi4 knockdown caused a general increase in piRNA expression from the entire genomic locus that produces the *gypsy* initiator piRNA (Supplementary Figure S4A). This intriguing finding suggests that Piwi4 controls the expression of selected piRNA cluster transcripts, the mechanism of which requires further investigation.

We next assessed the effect of PIWI knockdown on viral responder piRNA levels. As expected, responder piRNA production from the *gypsy*-targeted viruses was reduced upon knockdown of genes encoding the ping-pong partners Ago3 and Piwi5 and, to a lesser extent, Piwi6 (Figure 3C), even despite moderate knockdown efficiency (Supplementary Figure S4B). No significant effects on viral RNA levels were observed for any of these PIWI knockdowns, indicating that reduced piRNA levels were not due to reduced viral replication (Supplementary Figure S4C). Higher levels of the *gypsy*-derived initiator piRNA (Figure 3A) likely explain the observed increase in responder piRNA production upon Piwi4 knockdown (Figure 3C). We obtained similar results for the reporter viruses that are targeted by the EVE-derived initiator piRNA. Efficient knockdown of the ping-pong partners Ago3 and Piwi5 resulted in a dramatic decline in responder piRNA production from these viruses, while Piwi6 knockdown had a moderate effect (Figure 3D, Supplementary Figure S4D). Again, no effects of PIWI knockdown on viral RNA replication were observed (Supplementary Figure S4E). Importantly, Piwi4 knockdown, which did not result in altered EVE initiator piRNA levels (Figure 3B), barely affected responder piRNA production (Figure 3D), suggesting that Piwi4 has no direct involvement in ping-pong amplification of responder piRNAs. This is in line with previous findings that Piwi4 does not associate with Ago3 and Piwi5 (44) and is not required for ping-pong amplification of piRNAs (24,45,46). The Piwi5-dependency of *gypsy* responder piRNA production was further validated in Piwi5 knockout U4.4 cells, derived from the closely related mosquito *Aedes albopictus* (Figure 3E, Supplementary Figure S4F). Moreover, as expected from their ping-pong dependent production, responder piRNAs were specifically bound to Ago3 (Figure 3F).

We next investigated base-pairing requirements for responder piRNA production by introducing mutations into the seed region (nt 2–8), and around the putative slice site (nt 10–11) of the *gypsy* piRNA target site (Figure 3G, Supplementary Figure S4G). Responder piRNA production was strongly depleted in viruses in which mutations were introduced in the seed sequence (Mut 1–3, Mut 4–6 and Mut 7–9) compared to a virus bearing the intact target site (*gypsy*-28U, Figure 3H), indicating that seed-based target recognition is required for efficient responder piRNA production. Similarly, introducing mismatches around the slice site (Mut 10–11, Mut 9–12, Mut 10–13, Mut 10–15, Mut 8–13 and Mut 7–14) resulted in strongly reduced responder piRNA production. As viral RNA levels are virtually unchanged between all viruses, reduced responder piRNA production cannot result from differences in the amount of available substrate (Supplementary Figure S4H). Weak responder piRNA production was observed in two seed mutants (Mut 1–3 and Mut 4–6) and a slice site mutant (Mut10-11), suggesting that low level slicing may occur even in the absence of full complementarity in the seed region or the slice site, in line with earlier findings in *Drosophila* (8), mice (47) and mosquitoes (48). Altogether, these data show that slicing by the ping-pong partners Ago3 and Piwi5 is required for the production of responder piRNAs from the viral reporter.

### Zuc-mediated endonucleolytic cleavage defines piRNA 3′ends

**Figure 4.**
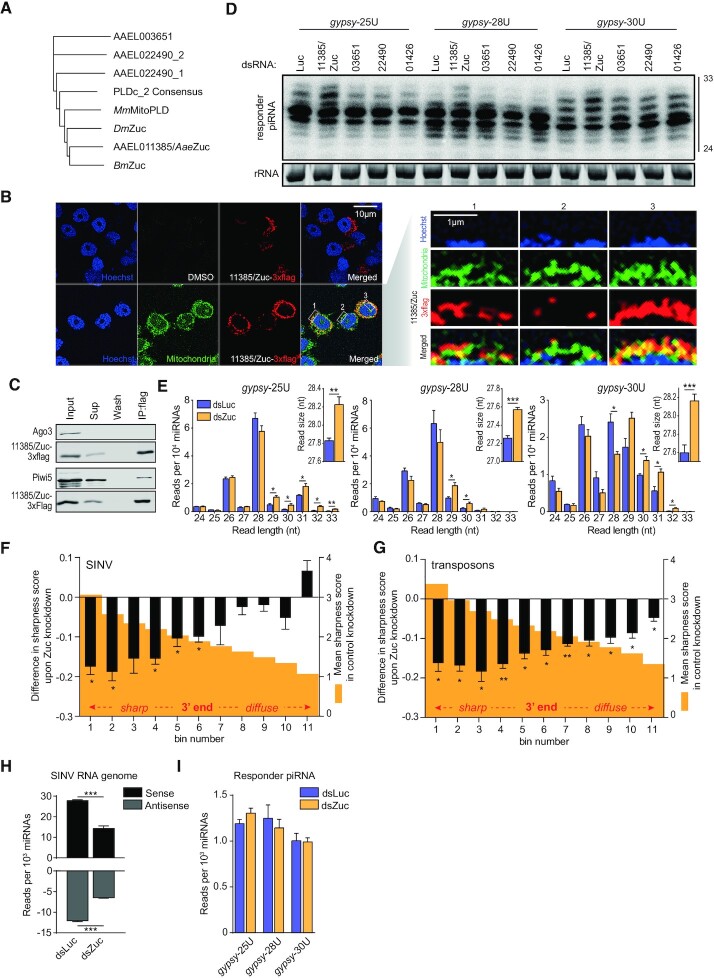
Zuc-mediated endonucleolytic cleavage defines vpiRNA 3′ends. (**A**) Phylogenetic tree based on PLDc_2 domains of *Ae. aegypti* PLDc_2 domain containing proteins and Zuc orthologs from *Drosophila (Dm*Zuc*)*, silkworm (*Bm*Zuc) and mouse (*Mm*MitoPLD). (**B**) Confocal microscopy images of Aag2 cells expressing 3 × flag tagged AAEL011385/Zuc. Mitochondria were stained using Mitoview green. Enlargements of the areas indicated by dashed boxes are shown in the right panels, with the nuclei oriented at the bottom. Scale bars denote 10μm (left panel) and 1μm (right panel). (**C**) Western blot of indicated proteins in AAEL011385/Zuc-3 × flag immunoprecipitation (IP). (**D**) Northern blot analysis of viral responder piRNAs in Aag2 cells infected with the indicated reporter viruses upon knockdown of *Ae. aegypti* PLDc_2 domain containing proteins and the PARN ortholog AAEL001426. Numbers indicate the VectorBase gene identifiers (without the AAEL0 prefix). The 24 and 33 nt size markers are inferred from an EtBr stained small RNA marker and rRNA stained by EtBr served as a loading control. The knockdown screen has been performed once; the AAEL011385/Zuc knockdown phenotype has been observed in eight independent infections. (**E**) Size distribution of viral responder piRNAs (SINV genome position 11585(+)) in small RNA sequencing libraries from Aag2 cells treated with dsRNA targeting luciferase (dsLuc) and Zuc (dsZuc). Read counts were normalized to the number of miRNAs in each library. The inset shows the average responder piRNA read size in Luc- and Zuc knockdown libraries. Bars and whiskers represent mean and SD of three independent libraries, respectively. Asterisks denote statistical significance as determined by unpaired two tailed t-tests with Holm-Sidak correction (* *P* < 0.05, ** *P* < 0.005, *** *P* < 0.0005). (**F**, **G**) Distribution of sharpness of piRNA 3′ ends upon Zuc knockdown. A sharpness score was attributed to the 275 most abundant viral piRNAs upstream of the artificial reporter cassette (F) and 4400 most abundant transposon piRNAs (G). The maximum score (3.81) is reached if 100% of piRNA reads that share the same 5′ end have the same length. For each piRNA, the sharpness score was determined in control (Luc) and Zuc knockdown conditions. The piRNAs were ranked and binned (*n* = 25 vpiRNAs and *n* = 400 transposon piRNAs per bin, respectively) according to the score in the control knockdown. For each bin, the mean sharpness score of all nine control knockdown libraries (three for each type of reporter virus) are plotted (orange shade, right y-axis). The difference of piRNA sharpness score upon Zuc knockdown was calculated and averaged for each type of reporter virus separately. Plotted is the mean and SEM of these average scores (left y-axis). A two-sided student's t-test was applied to each bin to assess whether its mean was significantly different from zero. * *P*< 0.05 and ** *P*< 0.005. (**H**) Read count of piRNAs mapping to the SINV genomic RNA in dsLuc and dsZuc treated Aag2 cells. Average piRNA counts were calculated from three independent libraries per virus infection. As the SINV genomic RNA is common for the three reporter viruses, these averages were used to determine mean piRNA read counts +/- SEM (bars and whiskers, respectively). Statistical significance determined by unpaired two tailed t-tests with HolmṇSidak correction is indicated with asterisks (*** *P* < 0.0005). (**I**) Responder piRNA levels in the reporter locus in dsLuc and dsZuc treated Aag2 cells infected with the indicated viruses. Bars and whiskers show the mean ± SD of three independent libraries.

The presence of sharply defined piRNA 3′ ends in combination with a bias for a downstream uridine (Figure 1) suggest that a Zuc-like endonuclease generates responder piRNAs 3′ ends and trailer piRNA 5′ ends. As the nuclease activity of Zuc lies in its phospholipase D (PLDc_2)-domain (39,40), we aligned the sequences of all *Ae. aegypti* PLDc_2-domain containing proteins with those of Zuc orthologs from fruit flies, silkworm and mouse (*Dm*Zuc, *Bm*Zuc and *Mm*MitoPLD, respectively) and found that AAEL011385 had the highest similarity to the various Zuc orthologs (Figure 4A). The protein encoded by this gene contains a fully conserved catalytic H(X)K(X4)D (HKD)-motif (Supplementary Figure S5A), suggesting that it is a functional endonuclease. Moreover, akin to Zuc orthologs in various other species (4,5), AAEL011385/Zuc localized to the mitochondria in Aag2 cells (Figure 4B). In *Drosophila*, a strong interaction between Zuc and Aub (the ortholog of Piwi5), as well as weak associations between Zuc and Ago3/Piwi have been observed (49–51). We thus evaluated whether AAEL011385/Zuc interacts with somatic *Aedes aegypti* PIWI proteins. While we readily detect Piwi5 in AAEL011385/Zuc immunoprecipitates, we did not observe interaction of AAEL011385/Zuc with Ago3 (Figure 4C), nor with Piwi4 and Piwi6 (Supplementary Figure S5B).

To our surprise, we found that AAEL011385/Zuc contains a sizeable insertion directly downstream of the catalytic HKD-motif (Supplementary Figure S5A). Moreover, during cloning of the AAEL011385/Zuc gene, we found that the size of this insertion is further increased by an additional 32 amino acids in Aag2 cells (Supplementary Figure S5A). Relative to mouse mitoPLD, *Drosophila and Bombyx* Zuc also contain (much smaller) insertions at the same position, which corresponds to the location of a helix that sticks out of core structure of *Drosophila* Zuc (40). Multiple sequence alignment revealed that a large insertion of >40 amino acids is present in *Culicidae* (mosquitoes) but not in other vector species such as ticks, lice, and tsetse flies, indicating that it is a variable region beyond *Aedine* mosquitoes, the function of which remains to be understood.

Using our viral piRNA reporter, we next aimed to validate AAEL011385 as the functional ortholog of *DmZuc*. Indeed, knockdown of AAEL011385/Zuc in Aag2 cells resulted in longer viral responder piRNAs with a broader size distribution (Figure 4D, Supplementary Figure S5C), which is consistent with less well-defined piRNA 3′ end generation. Knockdown of the other PLDc_2 domain containing proteins (AAEL003651 and AAEL022490) did not affect responder piRNA size (Figure 4D), despite very efficient knockdown (88–94% and 96–98% for AAEL003651 and AAEL022490, respectively, compared to 58–71% for AAEL011385/Zuc; Supplementary Figure S5D). Viral RNA levels were not consistently affected by knockdown of any of the genes tested (Supplementary Figure S5E). Small RNA deep-sequencing of dsAAEL011385/Zuc-treated Aag2 cells recapitulated the phenotype seen by northern blotting, with a general increase in piRNA length upon knockdown of AAEL011385/Zuc (Figure 4E). Based on these results, we conclude that AAEL011385 is the functional Zuc ortholog in *Ae. aegypti*.

We next studied the general effect of Zuc knockdown on the 3′ end sharpness on the entire population of vpiRNAs outside of the reporter cassette. Therefore, we defined sharpness scores for vpiRNAs that share the same 5′ end based on the Shannon entropy of their size distribution. A high score indicates that piRNAs with identical 5′ ends generally also had the same length whereas a lower score indicates a more diffuse size distribution. As expected, Zuc knockdown significantly reduced sharpness scores of vpiRNAs, in particular for those that had the sharpest 3′ ends in the control knockdown and were therefore likely the most dependent on Zuc cleavage (Figure 4F). The same effect was observed for piRNAs that mapped to transposon sequences (Figure 4G). Moreover, Zuc knockdown resulted in an increase in size of piRNAs produced from substrates of various origins, including transposons, mRNAs and viral RNAs (Supplementary Figure S5F), suggesting that Zuc is important for maturation of piRNAs from a broad repertoire of RNA substrates.

We next assessed the effect of Zuc depletion on overall vpiRNA levels. As expected, Zuc depletion reduced overall vpiRNA production from the SINV genomic and subgenomic RNA, which was common to the *gypsy*-25U, -28U and -30U viruses (Figure 4H). Yet, the abundance of the *gypsy*-triggered responder piRNA produced from the artificially introduced reporter locus was not affected by Zuc knockdown (Figure 4I), suggesting that an alternative mechanism contributes to 3′ end formation of this particular piRNA. We propose that upon Zuc knockdown, the Ago3-bound piRNA precursor is cleaved downstream of the uridine residue, either by a hitherto unknown endonuclease or by other PIWI-piRNA ribonucleoprotein complexes, as previously reported in *Drosophila* (11).

### A subset of responder piRNAs undergoes Nibbler-mediated trimming

**Figure 5.**
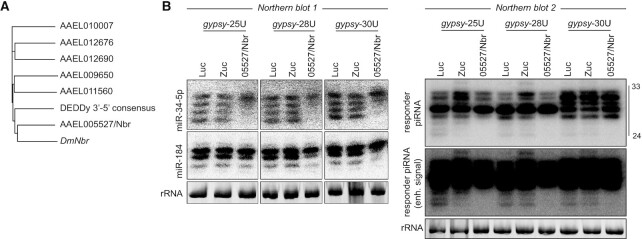
A subset of responder piRNAs undergoes Nbr-mediated trimming. (**A**) Phylogenetic tree based on the DEDDy 3′–5′ exonuclease domains identified in *Ae. aegypti* genes, along with the DEDDy consensus sequence and the DEDDy domain of *DmNbr*. (**B**) Northern blot analyses of miR-34-5p and miR-184 (left) and the viral responder piRNA (right) in Aag2 cells upon knockdown of Zuc and Nbr or control knockdown (Luc). RNA from the same knockdown experiment was analyzed on two separate northern blots. Ethidium bromide stained rRNA serves as a loading control. The Nbr knockdown phenotype has been observed in four independent infections.

In *Drosophila*, piRNA 3′ ends are generated by the concerted activities of two enzymes: the endonuclease Zuc (6–8) and the 3′–5′ exonuclease Nbr (11–13). We set out to identify the functional *Ae. aegypti* Nbr ortholog by predicting all DEDDy-type 3′ - 5′ exonuclease domain-containing proteins, which were used in a phylogenetic analysis along with *Drosophila* Nbr (*DmNbr)*. This analysis identified AAEL005527 as a one-to-one ortholog of *DmNbr* (Figure 5A). In addition, a recent study verified that AAEL005527 exhibits Mn^2+^-dependent, ssRNA-specific 3′–5′ exonuclease activity (52). To evaluate the role of trimming for the formation of responder vpiRNA 3′ ends in *Ae. aegypti*, we combined AAEL005527/Nbr knockdown with SINV infection using the *gypsy*-targeted reporter viruses. Knockdown was efficient (92–93%, Supplementary Figure S6A) and did not have a reproducible effect on viral RNA levels (Supplementary Figure S6B). Aside from its role in piRNA 3′ end formation, Nbr is required for trimming of microRNAs (miRNAs), including miR-34–5p (53,54). Thus, to verify that AAEL005527 is indeed the functional orthologue of *Drosophila* Nbr, we first assessed the effect of its depletion on trimming of two miRNA with heterogeneous 3′ ends in *Ae. aegypti*: miR-34–5p and miR-184 (55,56). Indeed, knockdown of AAEL005527 resulted in a marked reduction of miR-34–5p trimming. Similarly, AAEL005527 knockdown resulted in a specific decrease of smaller miRNA-184 isoforms (Figure 5B, northern blot 1), confirming that AAEL005527 is indeed the functional ortholog of *Drosophila* Nbr.

Next, we analyzed the effects of Nbr and Zuc knockdown on viral responder piRNA size. Similar to our previous findings, knockdown of Zuc resulted in an electrophoretic shift of viral responder piRNAs towards higher sizes (Figure 5B, northern blot 2). Interestingly, for all viruses tested, Nbr knockdown resulted in a reduction, specifically of shorter (<27 nt), responder piRNA isoforms, without affecting the larger isoforms (Figure 5B, northern blot 2, Supplementary Figure S6C). A similar reduction of shorter piRNA isoforms upon Nbr knockdown has previously been observed in *Drosophila* (12,13). These findings suggest that mosquito Nbr trims pre-piRNAs generated through a Zuc-mediated endonucleolytic cut, but that only a minor fraction of such pre-piRNAs undergo trimming.

The 3′−5′ exonucleases PNLDC1 and PARN-1 are responsible for trimming of piRNA 3′ ends in *B. mori* and *C. elegans*, respectively (57,58). While PNLDC1 is not conserved in *Ae. aegypti* (11), a clear mosquito ortholog of PARN-1 can be identified: AAEL001426. Knockdown of this gene however, had no effect on responder piRNA 3′ end formation in our viral reporter system (Figure 4D, Supplementary Figure S5C). In summary, we identified *Aedes aegypti* Nibbler to be involved in trimming of piRNAs and miRNAs.

### Targeting by an endogenous piRNA triggers trailer piRNA production

Endogenous piRNAs in *Ae. aegypti* show strong signatures of phased piRNA production (6); yet, it is unknown whether RNA from cytoplasmic viruses is processed similarly through piRNA phasing. This is especially interesting as the genomes of *Ae. aegypti* and *Ae. albopictus* mosquitoes contain a high number of endogenous viral elements (EVEs). These non-retroviral sequence elements are enriched in piRNA clusters and, accordingly, give rise to abundant piRNAs (28,31–33), which may guide the slicing of cognate RNA from acutely infecting viruses. It has recently been shown that EVE-derived piRNAs are indeed able to target cognate viruses and inhibit their replication (34,35), yet, it remains unknown whether piRNA phasing can expand the vpiRNA sequence repertoire after an initial cleavage by an endogenous piRNA.

**Figure 6.**
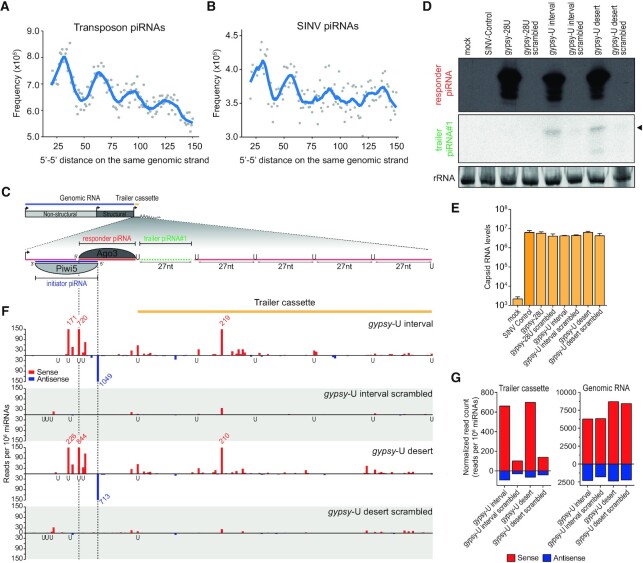
Targeting of viral RNA by an endogenous piRNA triggers trailer piRNA production. (**A**, **B**) Frequency distribution of piRNA 5′–5′ end distances for transposon (A) and SINV-derived (B) piRNAs. For each piRNA 5′ end, the number of downstream piRNAs 5′ ends is counted in a window of 20−150 nt. The combined distance frequencies are plotted and the curve is smoothened with local regression (LOESS). To reduce noise, only 5′ ends with a minimal read count of twenty were included in the analysis. (**C**) Schematic overview of the *gypsy*-U interval reporter virus. The inset shows a magnification of the non-coding reporter RNA expressed under control of the duplicated subgenomic promoter. This reporter RNA contains a target site for a Piwi5-bound *gypsy*-derived initiator piRNA, which guides the production of an Ago3-associated responder piRNA. The downstream sequence makes up the trailer cassette and either contains regularly spaced uridine residues (*gypsy-*U interval) or is devoid of uridine residues (*gypsy-*U desert). The responder piRNA and the first trailer piRNA are indicated in red and green, respectively. (**D**) Northern blot analysis of the responder and the first trailer piRNA (indicated by an arrowhead) produced from the indicated viruses. As a control, the target site was scrambled to abolish targeting by the *gypsy*-derived piRNA. The remainder of the responder piRNA site and the trailer cassette are identical to the respective non-scrambled U interval and U desert viruses. As additional controls, a virus bearing an intact target site, but no trailer cassette (gypsy-28U), and a virus that contains no insert (SINV Control) were used. rRNA stained with EtBr serves as a loading control. (**E**) RT-qPCR analyses of viral capsid RNA levels in Aag2 cells infected with the indicated viruses. Bars and whiskers show the mean and SD of three independent biological replicates. Unpaired two tailed t-tests with Holm-Sidak correction were used to determine statistically significant differences compared to SINV Control. (**F**) Normalized counts (in reads per 10^6^ miRNAs) of piRNA 5′ ends mapping to the initiator piRNA target site and trailer cassette of the indicated viruses. 5′ ends of initiator and responder piRNAs are indicated by dashed lines. Numbers in red and blue indicate counts that exceed the range of the y-axis. The position of uridine residues on the sense strand is indicated below the x-axis. Per condition, a single small RNA sequencing library was analyzed (same for G). (**G**) Total number of normalized sense (red) and antisense (blue) piRNA-sized (24–33 nt) reads mapping to the trailer cassette (left) and genomic RNA (right) of the indicated viruses. The areas of virus denoted as the trailer cassette and genomic RNA are shown in yellow and blue in (C).

We first confirmed that we could detect signatures of piRNA phasing in our small RNA deep-sequencing data. In line with prior findings (6), we observed that the distance between transposon-derived piRNA 5′ ends was regularly phased in intervals of ∼30 nt (Figure 6A). Strikingly, a similar periodicity was observed when we analyzed SINV-derived piRNAs (Figure 6B), indicating that viral RNA is also subjected to piRNA phasing. We noted that, compared to transposon-derived piRNAs, the periodicity of phasing of vpiRNAs is noisier after the second interval. This is likely explained by technical limitations of analyzing phasing signatures on the relatively small sequence of the SINV genome (approximately 11 kB in size).

Since vpiRNAs were biased for downstream uridines (Figure 1D, Supplementary Figure S1B) and positioning of uridine residues directed the 5′ end formation of the first trailer piRNA in our reporter viruses (Figure 2C), we next assessed the contribution of uridine residues to piRNA phasing. To this end, we introduced an additional non-coding RNA sequence downstream of the *gypsy* and EVE initiator piRNA target sites, which we termed the trailer cassette. To direct sequential Zuc-mediated endonucleolytic cleavage, this cassette contained uridine residues at regularly spaced 27 nt intervals in an RNA sequence that was otherwise devoid of uridines (U interval viruses, schematically shown in Figure 6C and Supplementary Figure S7C). As a control, these uridines were replaced by adenosine residues to create a trailer cassette completely devoid of uridines (U desert viruses). In Aag2 cells infected with these reporter viruses, but not control viruses in which the initiator piRNA target site was scrambled, responder piRNAs are abundantly produced (Figure 6D and Supplementary Figure S7A). In cells infected with the *gypsy*- and EVE-U interval virus, we also detected the first trailer piRNA using northern blotting (Figure 6D, Supplementary Figure S7A). Interestingly, we also observed the first trailer piRNA in cells infected with the U desert viruses (Figure 6D, Supplementary Figure S7A), suggesting that a downstream uridine residue was not essential for the maturation of the 3′ end of the first trailer piRNA. Importantly, in cells infected with viruses lacking the entire trailer cassette (*gypsy*-28U or EVE25/28U), responder piRNAs but no trailer piRNAs are detected, indicating that trailer piRNAs are specifically derived from the trailer cassette (Figure 6D, Supplementary Figure S7A). Moreover, no trailer piRNAs were produced in cells infected with control viruses containing a scrambled target site (Figure 6D, Supplementary Figure S7A), indicating that trailer piRNA production depends on initial targeting by the endogenous piRNA. Of note, differences in piRNA abundance were not the result of changes in viral RNA levels, which was similar for all viruses used (Figure 6E, Supplementary Figure S7B).

To assess phased piRNA biogenesis beyond the first trailer piRNA, we sequenced small RNAs produced in Aag2 cells infected with interval and desert viruses, as well as their respective scrambled control viruses. Mapping piRNAs onto the trailer cassette reveals production of additional piRNAs in cells infected with the *gypsy* and EVE initiator piRNA targeted viruses (Figure 6F, Supplementary Figure S7D). In contrast, barely any piRNAs mapping to the trailer cassette were recovered in cells infected with viruses bearing a scrambled target site (Figure 6G, left panel, Supplementary Figure S7E, left panel). Viral piRNA production from the SINV genome upstream of the artificial reporter and trailer cassettes was unaltered (Figure 6G, right panel, Supplementary Figure S7E, right panel), indicating that there are no differences in sensitivity of these viruses for processing by the vpiRNA biogenesis machinery. As both the pattern and level of piRNA production is highly similar between U interval and U desert viruses (Figure 6F and G, left panel, Supplementary Figure S7D and E, left panel), the presence of uridine residues to guide Zuc-mediated endonucleolytic cleavage appears to be dispensable for trailer piRNA production in the context of the artificial trailer cassette. Importantly, however, these data show that initial targeting by a genome-encoded piRNA results in the production of additional piRNAs downstream of the target site, resulting in diversification of the viral piRNA pool.

## CONCLUSION

Altogether, our results indicate that during acute infection with a cytoplasmic RNA virus, endogenous piRNAs can initiate piRNA production from viral genomic RNA via the ping-pong amplification loop (Supplementary Figure S8). The endonucleolytic and exonucleolytic activities of Zuc and Nbr, respectively are involved in maturation of the 3′ ends of piRNAs. Importantly, cleavage of viral RNA by an endogenous piRNA triggers the production of trailer piRNAs from the downstream cleavage fragment, thereby diversifying the piRNA sequence repertoire. These findings indicate that a few cleavage events by individual genome-encoded piRNAs are sufficient to launch a piRNA response that may eventually become independent of an endogenous trigger.

## DATA AVAILABILITY

Small RNA sequencing data has been deposited to the NCBI Sequence Read Archive under accession number SRP272125.

## Supplementary Material

gkab640_Supplemental_File

## Notes

Present address: Joep Joosten, Department of Synthetic Organic Chemistry, Institute for Molecules and Materials, Radboud University, Nijmegen, the Netherlands.
