# Supplementary material for: Endogenous piRNA-guided slicing triggers responder and trailer piRNA production from viral RNA in Aedes aegypti mosquitoes
Source: Nucleic Acids Res. 2021 Jul 31;49(15):8886–99. doi: 10.1093/nar/gkab640 (PMC8421150; doi:10.1093/nar/gkab640)
Supplement: gkab640_Supplemental_File [file gkab640_supplemental_file.pdf]

SUPPLEMENTAL INFORMATION

for

Endogenous piRNA-guided slicing triggers responder and trailer piRNA production from viral RNA in *Aedes aegypti* mosquitoes

Joep Joosten[1], Gijs J. Overheul[1], Ronald P. Van Rij[1+], and Pascal Miesen[1+]

[1]Department of Medical Microbiology, Radboud Institute for Molecular Life Sciences, Radboud University Medical Center, Nijmegen, P.O. Box 9101, 6500 HB, The Netherlands
[+] corresponding authors: ronald.vanrij@radboudumc.nl; pascal.miesen@radboudumc.nl

Supplemental text

An EVE-derived piRNA has the potential to trigger responder piRNA production.

Besides the *gypsy* initiator piRNA triggered reporter virus described in the main text, we generated a second set of viruses bearing target sites for an abundant Piwi5-associated piRNA derived from an EVE of flaviviral origin (described as FV53 in (1), Figure S2A, C). As the target site for the EVE-derived initiator piRNAs can be recognized by two piRNA isoforms that align at their 3' end and differ 3 nt in size (27 and 30 nt), target cleavage may result in the production of two Ago3-bound responder piRNAs isoforms that have an offset of 3 nt (blue shaded inset in Figure S2A).

Similar to the *gypsy* target site bearing viruses (Figure 2B), responder piRNA 3' end sharpness is moderately reduced as a function of increased distance between the initiator piRNA cleavage site and the first downstream uridine residue (Figure S2D), suggesting a role for exonucleolytic trimming in responder piRNA maturation.

For all viruses tested here, we observe abundant responder piRNAs as well as the production of a first trailer piRNA (indicated with yellow arrowheads in Figure S2E). As the trailer piRNA 5' ends perfectly overlap with the uridine residue, we propose that this residue guides cleavage of the viral RNA, simultaneously generating the responder pre-piRNA 3' end and the trailer piRNA 5' end, as also observed for the *gypsy* targeted viruses (Figure 2E).

Aside from these responder and trailer piRNAs, additional abundant sense piRNAs are produced from the sequence upstream of the initiator piRNA slice site (Figure S2E), suggesting that other, partially complementary, initiator piRNAs may target the sequence and instruct production of these additional sense piRNAs.

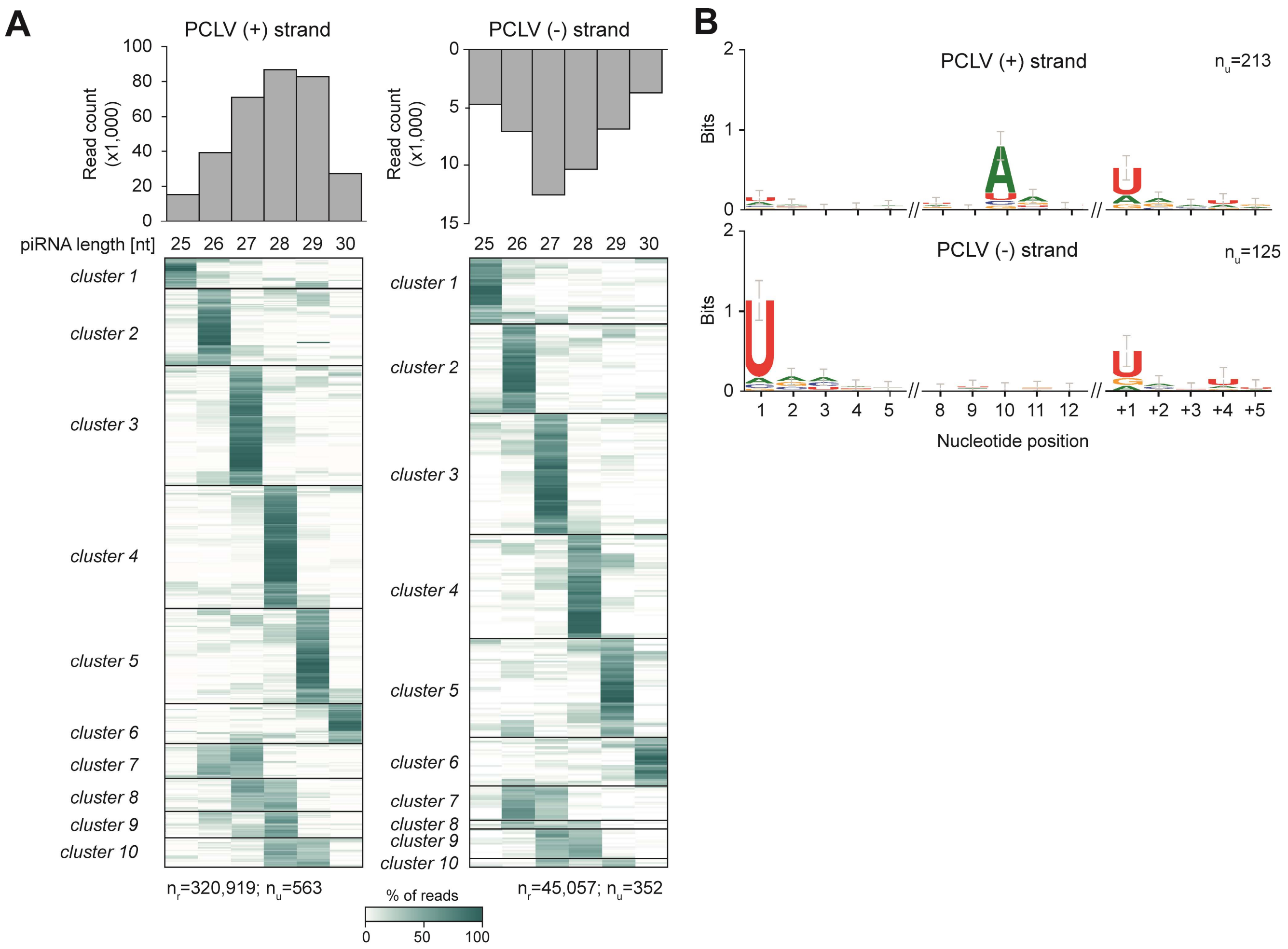

Figure S1. PCLV-derived piRNAs have sharp 3' ends

(A) Analysis of lengths of individual piRNAs derived from Phasi Charoen-like virus (PCLV). Data were generated from the combined three dsLuc small RNA libraries published in (2). Size profiles of piRNAs mapping to the sense ((+), left) and antisense ((-), right) strand of the virus genome are shown in the top panels. The heat maps show the relative size distribution of PCLV piRNAs that share the same 5' end (one 5' end is one line in the heat map). Shades of green indicate the percentage of reads contributing to the indicated piRNA length, white represents absence of reads of the specific size. The number of reads (n_r) and the number of unique piRNAs 5' ends (n_u) that underlie the heat map are indicated. A minimum of 20 reads per unique piRNA position was required to be included in the analysis. **(B)** Nucleotide biases for the indicated nucleotide positions of PCLV-derived vpiRNAs and the genomic region downstream (+1 to +5) of the vpiRNAs. Only vpiRNAs from (A) that had a dominant length (supported by at least 75% of reads) were considered in this analysis and all reads were collapsed to unique sequences, irrespective of read count. n_u indicates the number of sequences underlying the sequence logo.

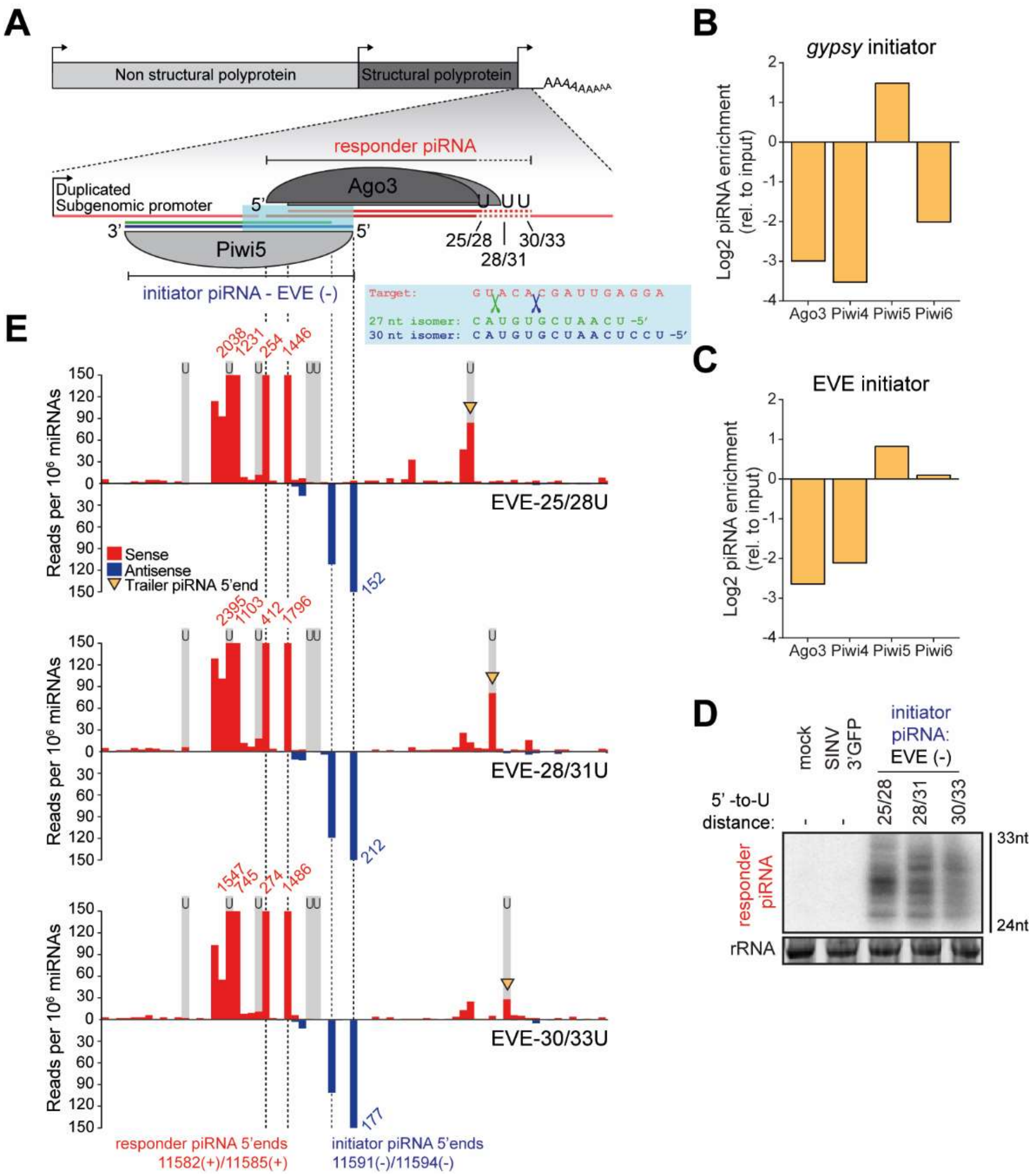

Figure S2. An EVE-derived piRNA triggers responder piRNA production from an acutely infecting RNA virus

(A) Schematic depiction of the SINV-based viral reporter system in which responder piRNA production is triggered by EVE initiator piRNAs. As the Piwi5-associated EVE initiator piRNA is expressed as two isoforms that align at their 3' end (blue and green lines), endonucleolytic cleavage may generate two Ago3-bound responder piRNAs (red lines) with a 5' end offset of 3 nt . The blue shaded inset depicts an enlargement of a part of the viral target RNA (red), the 5' ends of the two EVE-piRNA isoforms (27-nt: green and 30-nt: dark blue); the green and dark blue scissors represent their respective slice sites. **(B-C)** Log2 fold enrichment of the *gypsy* (B) and EVE (C) initiator piRNAs in PIWI protein immunoprecipitation (IP) small RNA libraries. Per condition, a single sequencing library previously described in (3,4) was analyzed. **(D)** Northern blot analyses of responder piRNAs produced in Aag2 cells infected with indicated EVE reporter viruses. The distance between responder piRNA 5' ends and the first downstream uridine residue is indicated. 24 and 33 nt size markers are inferred from EtBr staining of a small RNA marker, and EtBr stained rRNA serves as loading control. The northern blot is representative of three independent biological replicates. **(E)** Visualization of 5' ends of sense (red) and antisense (blue) piRNAs (24-33 nt) mapping to the non-coding reporter RNA sequence. Predicted initiator and responder piRNA 5' ends are indicated by dashed lines and positions of uridine residues on the sense strand are denoted by light grey shading. Red and blue numbers indicate read counts at positions where they exceed the y-axis range and yellow arrowheads indicate 5' ends of putative trailer piRNAs. A single sequencing library was analyzed for each virus infection.

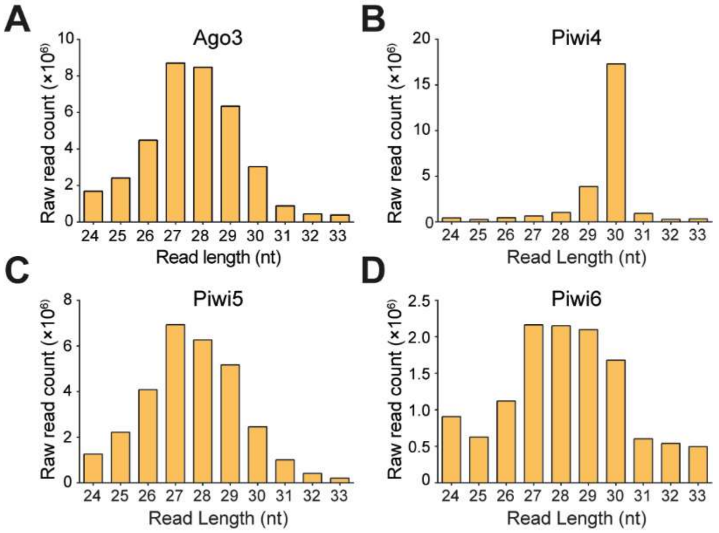

Figure S3. Size profiles of piRNAs associated with somatic PIWI proteins

(A-D) Size distribution of piRNA-sized (24-33 nt) small RNA reads in Ago3 (A), Piwi4 (B), Piwi5 (C), and Piwi6 IP (D) small RNA sequencing libraries. Piwi4 IP is dominated by two highly abundant piRNAs (tapiR1 and tapiR2 of 30 and 29 nt in size, respectively) that are involved in the degradation of maternally provided transcripts during embryonic development (4). Counts are raw, unmapped reads from our previously published small RNA sequencing data from endogenous PIWI protein IPs. (3,4). For each IP, a single sequencing library was analyzed.

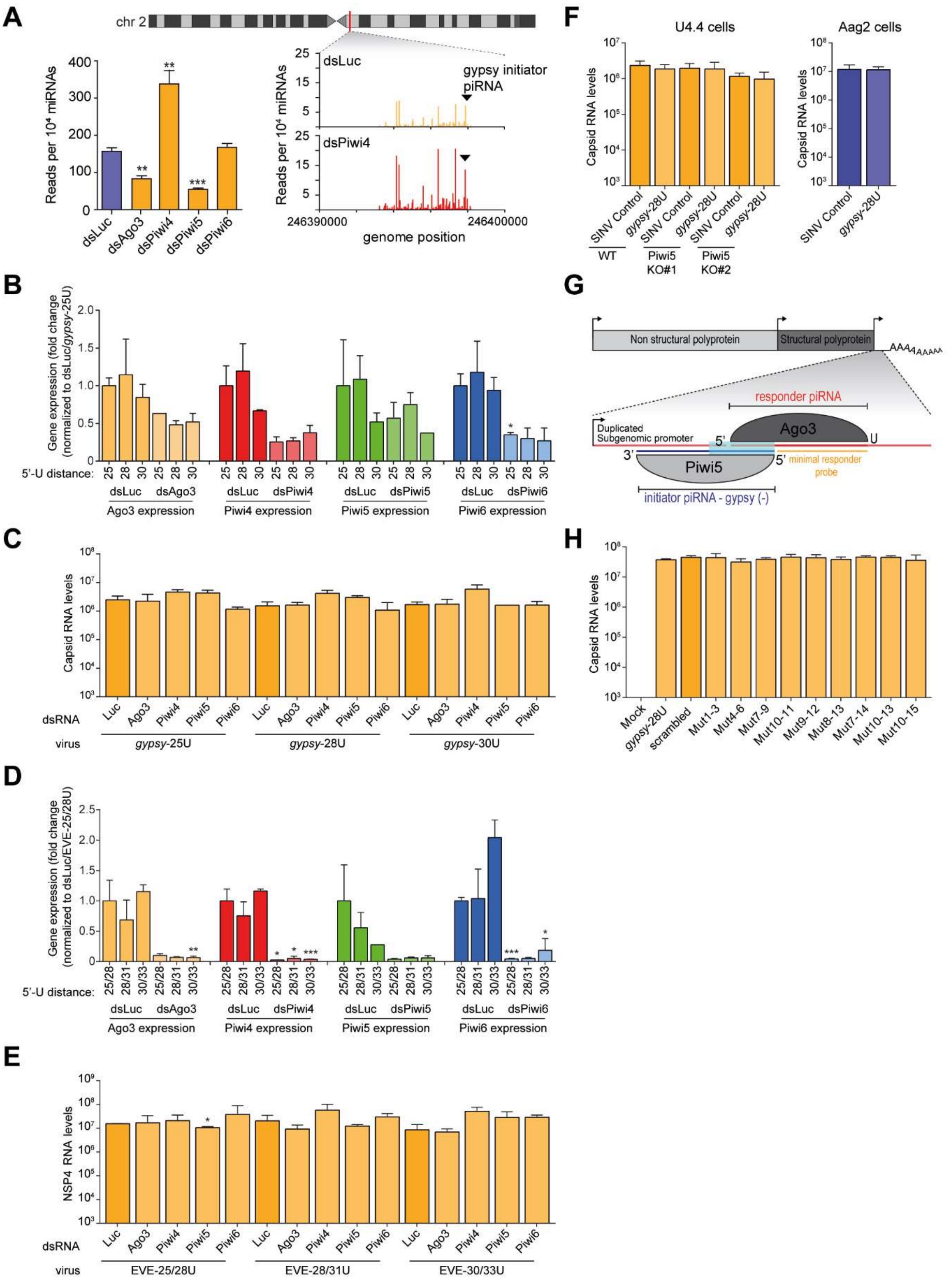

Figure S4. PIWI-knockdown does not affect virus replication

(A) piRNA abundance at the genomic locus from which the gypsy initiator piRNA is derived. The bar chart indicates normalized piRNA counts in PIWI knockdown libraries compared to a control (dsLuc) knockdown published in (2). The line graph shows the genome distribution of piRNAs at chromosome 2 from position 246,390,000 to 246,400,000 for the control and Piwi4 knockdown datasets analyzed in the bar chart. The mean read count of thee libraries is shown. The black arrowhead indicated the position of the gypsy initiator piRNA that triggers responder piRNA production in the reporter viruses. **(B-C)** RT-qPCR analyses of relative PIWI gene expression (B) and subgenomic capsid RNA levels (C) in the samples used in Figure 3C. PIWI gene RNA levels are shown as a fold change relative to dsLuc treated cells infected with *gypsy*-25U. **(D-E)** RT-qPCR analysis of PIWI knockdown efficiency (D) and genomic NSP4 RNA levels in the samples used in Figure 3D. **(F)** RT-qPCR analyses of capsid RNA levels in the indicated U4.4 (yellow) and Aag2 (blue) samples used in Figure 3E. **(G)** Schematic representation of the *gypsy*-28U virus that was used to study the effect of target site mutations on responder piRNA production (Figure 3G-H). The sequence in which seed and slice site mutations were introduced is indicated by light blue shading and the 'minimal responder' probe used to detect responder piRNAs in the northern blot of panel 3H is indicated in yellow. This minimal responder probe hybridizes to the last 18 nt of the 3' end of responder piRNAs, which are identical for all viruses. **(H)** RT-qPCR analysis of capsid RNA levels in samples used in Figure 3H. For all bar charts in this Figure, bars and whiskers show the mean and standard deviation (SD) of three independent biological replicates and unpaired two tailed t-tests with Holm-Sidak correction were performed for statistical analyses (* $P < 0.05$, ** $P < 0.005$, *** $P < 0.0005$).

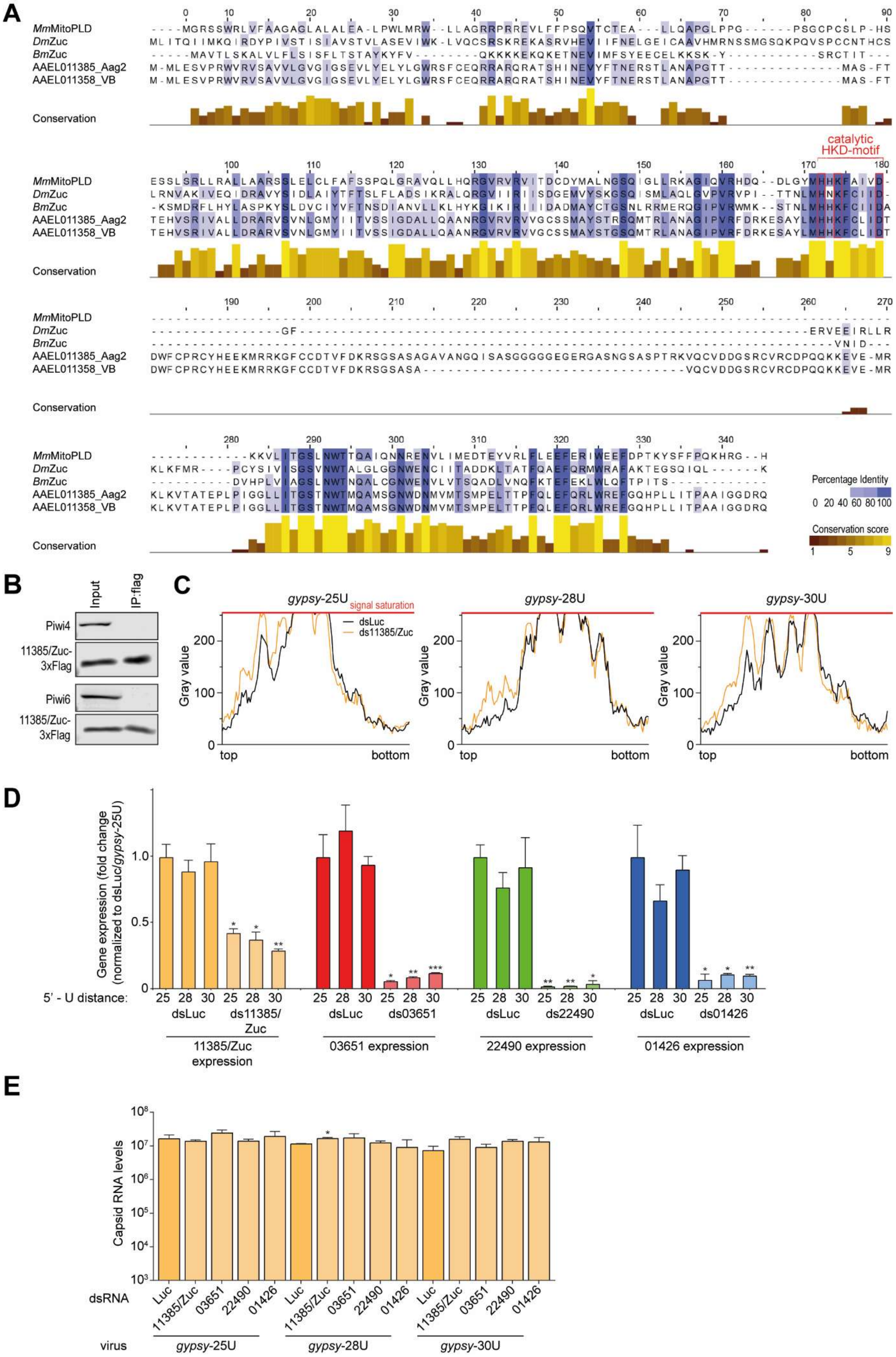
A
MmMitoPLD
DmZuc
BmZuc
AAEL011385_Aag2
AAEL011358_VB
Conservation
catalytic
HKD-motif
Percentage Identity
0 20 40 60 80 100
Conservation score
1 5 9
B
Input
IP:flag
Piwi4
11385/Zuc-3xFlag
Piwi6
11385/Zuc-3xFlag
C
gypsy-25U
signal saturation
dsLuc
ds11385/Zuc
gypsy-28U
gypsy-30U
Gray value
top
bottom
D
Gene expression (fold change (normalized to dsLuc/gypsy-25U)
5' - U distance:
25 28 30
dsLuc
ds11385/Zuc
11385/Zuc expression
ds03651
03651 expression
ds22490
22490 expression
ds01426
01426 expression
E
Capsid RNA levels
dsRNA
Luc
11385/Zuc
03651
22490
01426
virus
gypsy-25U
gypsy-28U
gypsy-30U

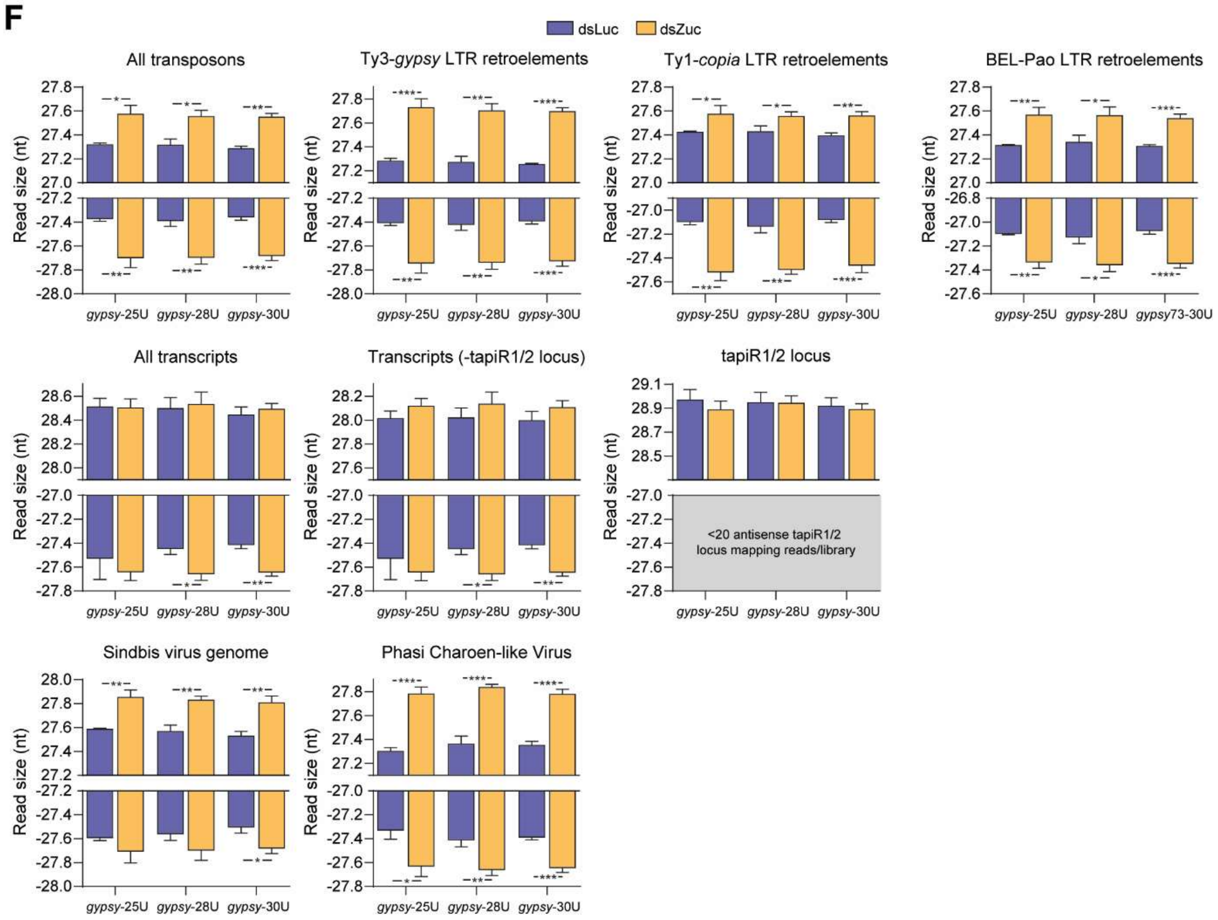

Figure S5. Mosquito Zuc is involved in 3' end formation of piRNAs from various substrates

(A) Multiple sequence alignment of Zucchini orthologs from *D. melanogaster* (*Dm*Zuc), *B. mori* (*Bm*Zuc) and *M. musculus* (*Mm*MitoPLD), and AAEL011385 as annotated in VectorBase (VB) and as sequenced from Aag2 cells. Residues shaded in blue are shared between ≥3 of the proteins, and the brown-yellow bars indicate conservation of physicochemical properties at each position. **(B)** Western blot analyses of Piwi4 and Piwi6 in the same Zuc-3xflag-IP material that was used in Figure 4C. **(C)** Quantification of the responder piRNA signal from the northern blot in Figure 4D, analyzed for the three *gypsy*-derived piRNA targeted viruses separately. The X-axis represents the position on the northern blot from top to bottom, the Y-axis shows responder piRNA signal intensity. **(D-E)** RT-qPCR analyses of the knockdown efficiency of the indicated genes (D) and viral capsid RNA levels (E). For (D), gene expression levels are depicted as fold changes relative to dsLuc treated cells infected with the *gypsy*-25U virus. Knockdown efficiencies and viral RNA levels were tested against dsLuc treated samples infected with the same virus. **(F)** Average size of piRNAs (24-33 nt) derived from the indicated substrates in small RNA sequencing libraries of dsLuc and dsZuc treated Aag2 cells. The average length of two piRNAs (tapiR1/2) involved in degradation of maternal transcripts during embryogenesis (4), is not affected by Zuc knockdown. As virtually no antisense reads map to the tapiR1/2 locus, these data are not shown. For all bar charts, bars and whiskers represent mean and SD of three independent replicates. Unpaired two tailed t-tests with Holm-Sidak correction for multiple comparisons were used to determine statistical significance (* $P < 0.05$, ** $P < 0.005$, *** $P < 0.0005$).

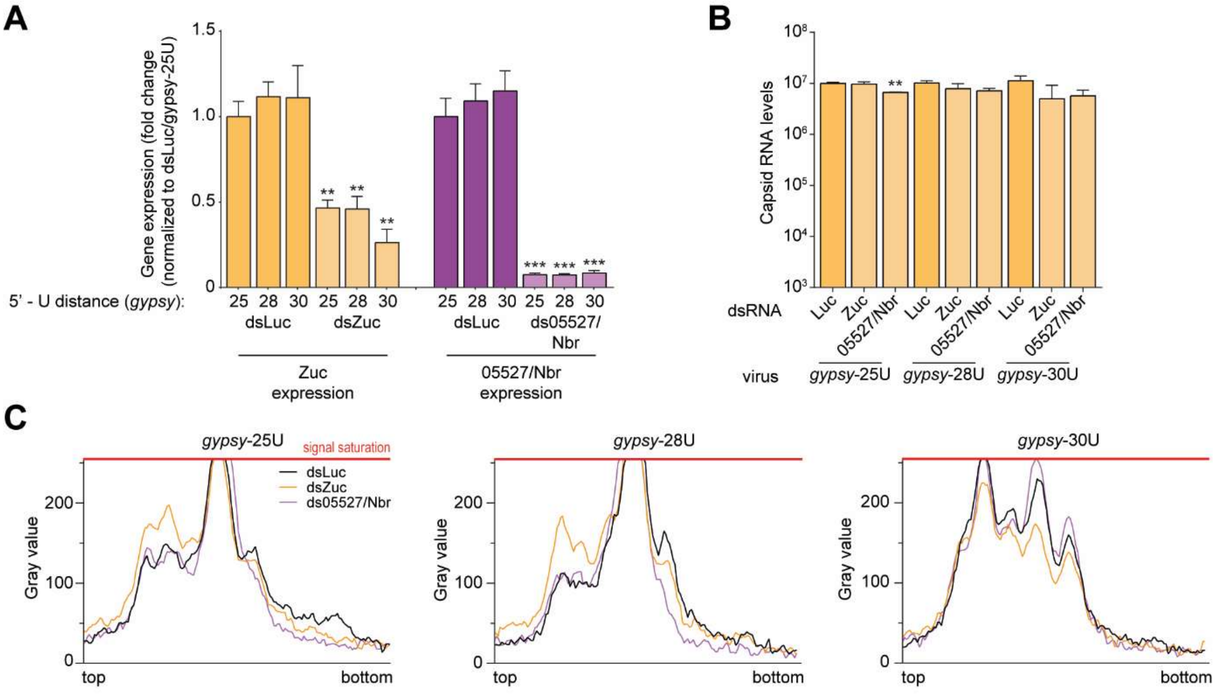

Figure S6. Nbr- and Zuc knockdown affect piRNA biogenesis

(A-B) RT-qPCR analyses of the knockdown efficiencies of indicated genes (A) and viral capsid RNA levels (B). Bars and whiskers represent the mean +/- SD of three independent biological replicates. For (A), expression values represent the fold change relative to dsLuc treated cells infected with the *gypsy*-25U virus. Unpaired two tailed t-tests with Holm-Sidak correction for multiple comparisons were used to determine statistical significance (** $P < 0.005$, *** $P < 0.0005$). Knockdown efficiencies and viral RNA copy numbers were tested against dsLuc treated Aag2 cells infected with the same virus. **(C)** Quantification of the viral responder piRNA signal on northern blot 2 in Figure 5B, showing the effect of Nbr knockdown on responder piRNA size during infection with the indicated viruses. The X-axis represents the position on the northern blot from top to bottom, the Y-axis represents responder piRNA signal intensity.

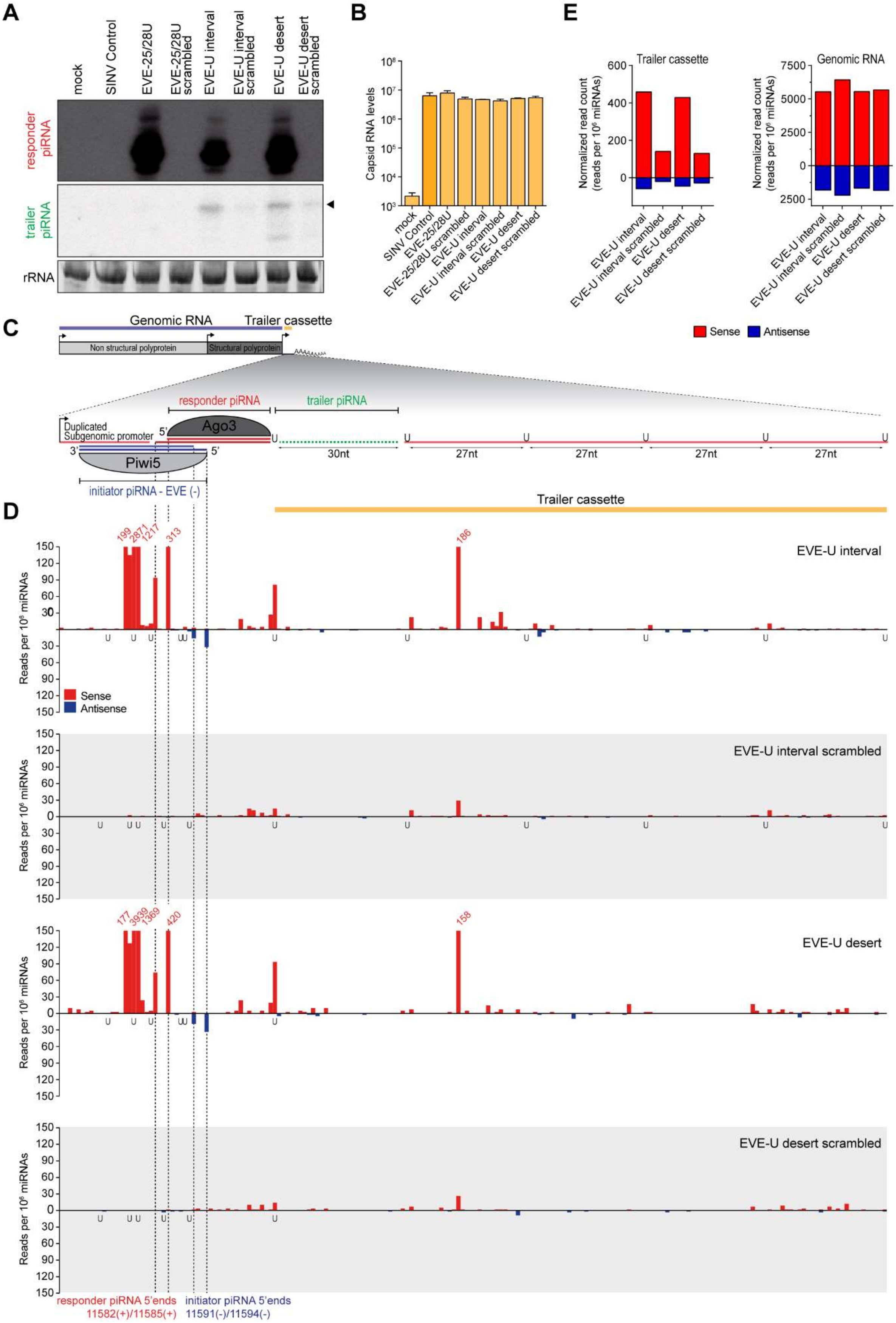

Figure S7. An EVE-derived piRNA triggers trailer piRNA production downstream of its target site

(A) Northern blot analyses showing the production of the responder piRNA and the first putative trailer piRNA (indicated by an arrowhead) from the indicated viruses. As a control, the target site was scrambled to abolish targeting by the EVE-derived initiator piRNA. The remainder of the responder piRNA site and the trailer cassette was identical to the U interval and U desert viruses. Additional controls include a virus that lacks the trailer cassette, but contains an EVE piRNA-target site (FV25-28) and a control virus without insert (SINV Control). A schematic overview of the EVE targeted interval virus is shown in (C). rRNA stained by EtBr serves as loading control. n = one biological replicate. **(B)** Capsid RNA levels in Aag2 cells infected with indicated viruses as determined by RT-qPCR. Bars and whiskers denote the mean and SD of three independent biological replicates, respectively. Unpaired two tailed t-tests with Holm-Sidak correction were used to compare differences with the control virus. **(C)** Schematic overview of the EVE interval virus. In this virus, a duplicated subgenomic promoter drives the expression of a non-coding reporter RNA (shown in the magnification), which contains a target site for two isoforms of a Piwi5-associated EVE-derived initiator piRNA (shown in blue). Slicing of the reporter RNA in the ping-pong amplification

loop thus may give rise to two differently sized Ago3-associated responder piRNAs (shown in red). Located downstream of this responder piRNA is the trailer cassette, which either contains uridine residues at regularly spaced intervals (EVE-U interval) or is completely devoid of uridines (EVE-U desert). The first trailer piRNA, which was detected in (A), is shown in green. **(D)** Normalized counts of 5’ ends of sense (red) and antisense (blue) piRNA-sized reads (24-33 nt) mapping to the non-coding reporter RNA. The 5’ ends of the two EVE-derived initiator piRNA isoforms, as well as the 5’ ends of corresponding responder piRNAs are indicated by dashed lines. The position of uridine residues is indicated below the x-axis. The numbers in red indicate normalized read counts that exceed the range of the y-axis. Per condition, a single small RNA sequencing library was analyzed (same for (E)).**(E)** Quantification of the number of sense (red) and antisense (blue) piRNAs mapping to the trailer cassette (left) and genomic RNA (right) of the indicated recombinant Sindbis viruses. The areas of the viruses denoted as trailer cassette and genomic RNA are indicated in (C) in yellow and blue, respectively.

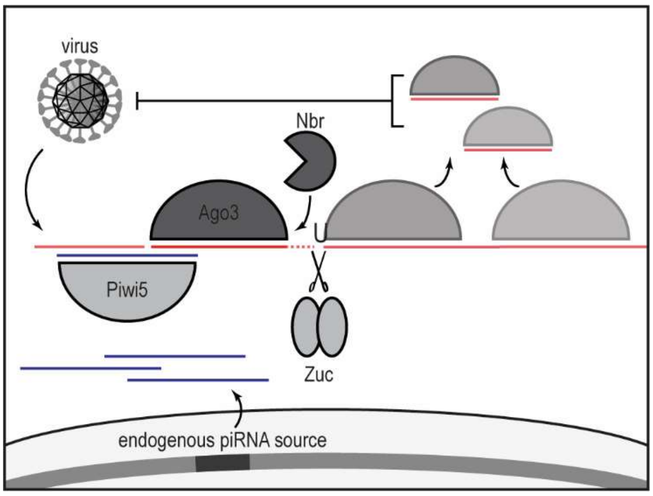

Figure S8. A model of piRNA-based adaptive immunity in mosquitoes

Genome-encoded endogenous viral elements (EVE) or other endogenous loci give rise to a pool of Piwi5-associated initiator piRNAs that have the potential to target newly infecting viruses. Upon infection with a virus containing a cognate sequence, EVE-derived piRNAs trigger the production of Ago3-bound responder piRNAs from the viral RNA, which are generated and maturated by the combined activities of Zuc and Nbr. In addition, targeting of the viral RNA by the ping-pong machinery initiates the processing of the cleavage fragment into additional downstream trailer piRNAs, thus expanding the piRNA sequence pool that is able to target viral RNA.

Supplemental materials and methods

Cell culture, dsRNA transfection and infection of Aag2 and U4.4 cells

Ae. aegypti Aag2 and *Ae. albopictus* U4.4 cells were grown in Leibovitz's L-15 medium (Invitrogen) supplemented with 10% fetal bovine serum (Gibco), 50 U/ml Penicillin, 50 µg/mL Streptomycin (Invitrogen), 1x Non-essential Amino Acids (Invitrogen) and 2% Tryptose phosphate broth solution (Sigma) at 25°C. Cell lines were maintained by splitting twice weekly according to confluency.

For knockdown experiments, ~1×10^6 cells were seeded in 6-well plates and allowed to attach for 16-24 hrs. Subsequently, cells were transfected with gene-specific dsRNA using X-tremeGENE HP DNA Transfection Reagent (Roche) according to the manufacturer's instruction, and re-transfected 48 hrs later to ensure sustained knockdown (750 ng dsRNA with 3 µL X-tremeGENE HP per well). Three hrs after the second transfection, cells were infected with indicated viruses at a multiplicity of infection (MOI) of 0.1 and RNA was harvested 72 hrs post infection. In experiments where no knockdown was performed, cells were infected 16-24 hrs after seeding at MOI = 0.1, followed by RNA extraction at 72 hrs post infection.

Generation of reporter viruses

To introduce the target sites for *gypsy*- and EVE-initiator piRNAs, as well as the responder reporter cassette, the pTE3'2J-GFP plasmid encoding the previously described SINV-GFP (5,6) was first digested using XbaI to remove the GFP-gene. Target sites and the reporter locus were subsequently introduced downstream of the duplicated subgenomic promoter by ligation of annealed oligonucleotides (see below), making use of the overhangs generated by the XbaI enzyme (initiator piRNA target site in red, downstream uridine position is indicated in **bold** font).

**Primer name**	**Nucleotide sequence (5' - 3')**
FW-EVE-25/28U	CTAGAGACCAGGACAGGCAATGAGACTGAGTACACGATTGAGGAGGCGCGAGCACAGAC**T**AGAAC<br>AGCAGAACGACGAGCAAAGGCGGCCGCAGCCT
RV-EVE-25/28U	CTAGAGGCTGCGGCCGCCTTTGCTCGTCGTTCTGCTGTTCTAGTCTGTGCTCGCGCCTCCTCAATC<br>GTGTACTCAGTCTCATTGCCTGTCCTGGTCT
FW-EVE-28/31U	CTAGAGACCAGGACAGGCAATGAGACTGAGTACACGATTGAGGAGGCGCGAGCACAGACCAG**T**AC<br>AGCAGAACGACGAGCAAAGGCGGCCGCAGCCT
RV-EVE-28/31U	CTAGAGGCTGCGGCCGCCTTTGCTCGTCGTTCTGCTGTACTGGTCTGTGCTCGCGCCTCCTCAATC<br>GTGTACTCAGTCTCATTGCCTGTCCTGGTCT
FW-EVE-30/33U	CTAGAGACCAGGACAGGCAATGAGACTGAGTACACGATTGAGGAGGCGCGAGCACAGACCAGAA**T**<br>AGCAGAACGACGAGCAAAGGCGGCCGCAGCCT
RV-EVE-30/33U	CTAGAGGCTGCGGCCGCCTTTGCTCGTCGTTCTGCTATTCTGGTCTGTGCTCGCGCCTCCTCAATC<br>GTGTACTCAGTCTCATTGCCTGTCCTGGTCT
FW-*gypsy*-25U	CTAGAGACCAGGACAAAGCGCACATACAGTGCAATGTCAGAGCAGGCGCGAGCACAGAC**T**AGAAC<br>AGCAGAACGACGAGCAAAGGCGGCCGCAGCCT
RV-*gypsy*-25U	CTAGAGGCTGCGGCCGCCTTTGCTCGTCGTTCTGCTGTTCTAGTCTGTGCTCGCGCCTGCTCTGAC<br>ATTGCACTGTATGTGCGCTTTGTCCTGGTCT
FW-*gypsy*-28U	CTAGAGACCAGGACAAAGCGCACATACAGTGCAATGTCAGAGCAGGCGCGAGCACAGACCAG**T**AC<br>AGCAGAACGACGAGCAAAGGCGGCCGCAGCCT
RV-*gypsy*-28U	CTAGAGGCTGCGGCCGCCTTTGCTCGTCGTTCTGCTGTACTGGTCTGTGCTCGCGCCTGCTCTGAC<br>ATTGCACTGTATGTGCGCTTTGTCCTGGTCT
FW-*gypsy*-30U	CTAGAGACCAGGACAAAGCGCACATACAGTGCAATGTCAGAGCAGGCGCGAGCACAGACCAGAA**T**<br>AGCAGAACGACGAGCAAAGGCGGCCGCAGCCT
RV-*gypsy*-30U	CTAGAGGCTGCGGCCGCCTTTGCTCGTCGTTCTGCTATTCTGGTCTGTGCTCGCGCCTGCTCTGAC<br>ATTGCACTGTATGTGCGCTTTGTCCTGGTCT

To introduce the U-interval and U-desert reporter cassettes, the *gypsy*-28U and EVE-25/28U viruses were digested using NotI followed by ligation of annealed oligo's (see below).

**Primer name**	**Nucleotide sequence (5' - 3')**
FW-U-interval	GGCCTCGCAAGCACAAAGCCGAAGCAGACGAG**T**CGAACCAGGCAAGCAAAGCGACAACGA**T**AAGC<br>AAAGCAACGGCAAAGCAAGCCAA**T**AACGAAAACAGCAAAGCAGACGAACAC**T**GC
RV-U-interval	GGCCGCAGTGTTCGTCTGCTTTGCTGTTTTCGTTATTGGCTTGCTTTGCCGTTGCTTTGCTTATCGTT<br>GTCGCTTTGCTTGCCTGGTTCGACTCGTCTGCTTCGGCTTTGTGCTTGCGA
FW-U-desert	GGCCACGCAAGCACAAAGCCGAAGCAGACGAGACGAACCAGGCAAGCAAAGCGACAACGAAAAGC<br>AAAGCAACGGCAAAGCAAGCCAAAAACGAAAACAGCAAAGCAGACGAACACAGC
RV-U-desert	GGCCGCTGTGTTCGTCTGCTTTGCTGTTTTCGTTTTTGGCTTGCTTTGCCGTTGCTTTGCTTTTCGTT<br>GTCGCTTTGCTTGCCTGGTTCGTCTCGTCTGCTTCGGCTTTGTGCTTGCGT

Subsequently, viruses were grown as described previously (7).

Generation of target site mutant viruses

Target site mutations were introduced into the plasmid encoding the *gypsy*-28U virus by mutagenesis PCR, using the primers shown below. PCR products were DpnI-treated and In-fusion (Takara Biotech) was used to circularize the plasmid for transformation. After verification of the sequence by Sanger sequencing, viruses were grown as described previously (7).

**Primer name**	**Nucleotide sequence (5' - 3')**
FW_mut1-3	GTCAGACGTGGCGCGAGC
RV_mut1-3	CGCGCCACGTCTGACATTGC
FW_mut4-6	AATGTCTCTGCAGGCGCGAG
RV_mut4-6	GCCTGCAGAGACATTGCACTGT
FW-mut7-9	TGCAATCAGAGAGCAGGCGC
RV-mut7-9	TGCTCTCTGATTGCACTGTATGTGC
FW-mut10-11	CAGTGCATAGTCAGAGCAGGCG
RV-mut10-11	TCTGACTATGCACTGTATGTGCGC
FW-mut9-12	CAGTGCTTACTCAGAGCAGGCGC
RV-mut9-12	TCTGAGTAAGCACTGTATGTGCGCTTTG
FW-mut8-13	CAGTGGTTACACAGAGCAGGCGCG
RV-mut8-13	TCTGTGTAACCACTGTATGTGCGCTTTGTC
FW-mut7-14	CAGTCGTTACAGAGAGCAGGCGCGAG
RV-mut7-14	TCTCTGTAACGACTGTATGTGCGCTTTGTCCTG
FW-mut10-13	CAGTGGTTAGTCAGAGCAGGCGC
RV-mut10-13	TCTGACTAACCACTGTATGTGCGCTTTGTC
FW-mut10-15	CAGACGTTAGTCAGAGCAGGCGCGA
RV-mut10-15	TCTGACTAACGTCTGTATGTGCGCTTTGTCCT

Using the method described above, scrambled *gypsy 73* and EVE target sites were introduced into the *gypsy*-28U, *gypsy*-U interval, *gypsy*-U desert, EVE-25/28U, EVE-U interval and EVE-U desert backbones, using primers listed below.

**Primer name**	**Nucleotide sequence (5' - 3')**
FW_*gypsy*-scrambled	ACCGAGAGTACCGCAGAACAACGGCGCGAGCACAGAC
RV_*gypsy*-scrambled	TGCGGTACTCTCGGTATAATCCTGTCCTGGTCTCTAGAGGTGG
FW_EVE-scrambled	GCGTATACAGGTAAGGCTCAAGGGCGCGAGCACAGAC
RV_EVE-scrambled	CTTACCTGTATACGCTCTACTTCGTCCTGGTCTCTAGAGGTGG

dsRNA production for knockdown experiments

Gene-specific PCR products bearing T7 promoter sequences at both ends were generated in one of two ways. Either the T7 promoter sequence was introduced directly with the gene-specific PCR, or a universal GC-rich tag was introduced in the first PCR, to which the T7 promoter sequence was added in a second PCR. Oligonucleotides used to create the T7-promoter tagged PCR products are shown below (T7 promoter sequence in **bold** font, the universal GC-rich tag in underlined font):

**Primer name**	**Nucleotide sequence (5' - 3')**
F-T7-Luc	**TAATACGACTCACTATAGGGAGA**TATGAAGAGATACGCCCTGGTT
R-T7-Luc	**TAATACGACTCACTATAGGGAGA**TAAAACCGGGAGGTAGATGAGA
F-T7-Ago3	**TAATACGACTCACTATAGGGAGA**TGCTTACTCGTGTCGCGTAG
R-T7-Ago3	**TAATACGACTCACTATAGGGAGA**GGCATGGCAGATCCAATACT
F-T7-Piwi4	**TAATACGACTCACTATAGGGAGA**CGTGGAAGTCCTTCTTCTCG
R-T7-Piwi4	**TAATACGACTCACTATAGGGAGA**TGTCAGTTGATCGCTTCTCAA
F-T7-Piwi5	**TAATACGACTCACTATAGGGAGA**GCCATACATCGGGTCAAAAT
R-T7-Piwi5	**TAATACGACTCACTATAGGGAGA**CTCTCCACCGAAGGATTGAA
F-T7-Piwi6	**TAATACGACTCACTATAGGGAGA**CAACGGAGGATCTTCACGAG
R-T7-Piwi6	**TAATACGACTCACTATAGGGAGA**AATCGATGGCTTGATTTGGA
F-GC-AAEL011385/Zuc	GCCCGACGCCAGTGCGTTTCGATCG
R-GC-AAEL011385/Zuc	CGCCTCGGCCCCGTAATGAGCAACCC
R-GC-AAEL003651	GCCCGACGCGTGGTGCCTGCTTCG
F-GC-AAEL003651	CGCCTCGGCTGCCACATTTGTCCTAAGG
F-GC-AAEL022490	GCCCGACGCTTTCGCCCTGTTTCCC
R-GC-AAEL022490	CGCCTCGGCGATCATCACGTATCGGTCC
F-GC-AAEL001426	GCCCGACGCGACGATTCGCCAAACC
R-GC-AAEL001426	CGCCTCGGCCCATTTTCCTCTTCTTTCG
R-GC-AAEL005527/Nbr	GCCCGACGCCCCCCGATAGTGAGCC
F-GC-AAEL005527/Nbr	CGCCTCGGCCCAAAGCAGCTGTAAATCC
F-T7-universal primer	**TAATACGACTCACTATAGGGAGA**GCCCGACGC

R-T7-universal primer	**TAATACGACTCACTATAGGGAGA**CGCCTCGGC

Subsequently, these PCR products were used as template for *in vitro* transcription by T7 polymerase for 4 hours at 37°C. Afterwards, the *in vitro* transcribed RNA was heated to 80°C and gradually cooled to room temperature to allow the formation of dsRNA, which was subsequently purified using the GenElute Mammalian Total RNA kit (Sigma).

Generation of Piwi5 knockout U4.4 cells

Description and characterization of Piwi5 knockout U4.4 cells is described elsewhere (manuscript in prep).

Phylogenetic analyses

The PFAM PLDc_2 domain (PF13091) consensus sequence was used as input for iterative searches against the *Ae. aegypti* proteome using JackHMMR (https://www.ebi.ac.uk/Tools/hmmer/search/jackhmmer) to identify all *Ae. aegypti* PLDc_2 domain containing proteins. To generate a neighbor-joining tree, the PLDc_2 domain sequences from the identified genes and the Zuc orthologs in *Drosophila*, silkworm and mouse were aligned using M-Coffee (http://tcoffee.crg.cat/apps/tcoffee/do:mcoffee). One of the identified genes, AAEL022490, contains two PLDc_2 domains, both of which were used as separate inputs in the subsequent analysis. The same approach using the DEDDy type 5'-3' exonuclease (cd06141) CDD-consensus sequence was used for the identification of *Ae. aegypti* Nbr.

For the alignment of full-length Zuc sequences, the entire sequence of *Ae. aegypti* Zuc as annotated on VectorBase (www.vectorbase.org), the Zuc sequence as determined by us from Aag2 cells and the sequences of Zuc orthologs in *Drosophila,* silkworm, mouse were aligned using M-Coffee with default settings and visualized using Jalview 2.11.0. Conservation of physicochemical properties at each position was analyzed in Jalview 2.11.0., and is based on the Analysis of Multiply Aligned Sequences (AMAS) method described in (8).

RNA isolation

Cells were lysed in 1 mL RNA-Solv reagent (Omega Bio-tek), followed by RNA extraction through phase separation and isopropanol precipitation. RNA integrity was evaluated on EtBr stained agarose gels, and RNA concentration was determined using the Nanodrop ND-1000.

RT-qPCR

For RT-qPCR analyses, DNaseI (Ambion)-treated RNA was reverse transcribed using the Taqman reverse transcriptase kit (Life Technologies) and PCR amplified in the presence of SYBR green, using the GoTaq qPCR system (Promega) according to the manufacturers' recommendations. In knockdown experiments, expression levels of target genes were normalized to the expression of the housekeeping gene lysosomal aspartic protease (LAP) for *Ae. aegypti* samples or Ribosomal Protein L5 (RpL5) for *Ae. albopictus* samples and fold changes were calculated using the $2^{-\Delta\Delta CT}$ method (9). Viral RNA levels were calculated using standard curves generated from a tenfold serial dilution series of a plasmid containing the SINV genome sequence. The standard curves were: $\log_{10}$ viral RNA level=-0.2041×CT + 10.572 (R^2=0.9983) for Capsid and $\log_{10}$ viral RNA levels=-0.3029×CT + 13.027 (R^2=0.9625) for Nsp4. The following primers were used:

**Primer name**	**Nucleotide sequence (5' - 3')**
qF-SINV-Capsid	CTGGCCATGGAAGGAAAGGT
qR-SINV-Capsid	CCACTATACTGCACCGCTCC
qF-SINV-Nsp4	AACTCTGCCACAGATCAGCC
qR-SINV-Nsp4	GGGGCAGAAGGTTGCAGTAT
qF-LAP	GTGCTCATTCACCAACATCG
qR-LAP	AACTTGGCCGCAACAAATAC
qF-*Aal*-RpL5	TCGCTTACGCCCGCATTGAGGGTGAT
qR-*Aal*-RpL5	TCGCCGGTCACATCGGTACAGCCA
qF-AAEL011385/Zuc	GATGACCCGTTTGGCC

qR-AAEL011385/Zuc	CATTGGCCACTGCACC
qF-AAEL003651	CCGTTGTGTTCAACAACC
qR-AAEL003651	GAGTTAACGGGACGTAATCC
qF-AAEL022490	CGGAATGCAGCATTCTGATG
qR-AAEL022490	GTTTCAGCTTTTCGTAGAATTTCC
qF-AAEL001426	GACACAACATGCTGTTGG
qR-AAEL001426	TGACGGCTTCGAACAC

Small RNA northern blotting

For small RNA northern blotting, 4-12 µg of RNA was size separated by denaturing urea polyacrylamide (15%) gel electrophoresis, transferred to nylon membranes and crosslinked using 1-ethyl-3-(3-dimethylaminopropyl)carbodiimide hydrochloride (EDC) crosslinking as described in (10). ^{32}P labelled DNA probes (sequences shown below) were used to detect small RNAs.

**Probe name**	**Nucleotide sequence (5' - 3')**
gypsy-responder:	TTCTGGTCTGTGCTCGCGCCTGCTCTGACA
EVE-responder:	TTCTGGTCTGTGCTCGCGCCTCCTCAATCG
Trailer piRNA:	CTCGTCTGCTTCGGCTTTGTGCTTGCG
miR34-5p:	CAACCAGCTAACCACACTGCCA
miR184:	GCCCTTATCAGTTCTCCGTCCA
minimal responder:	CTGGTCTGTGCTCGCGCC

To generate the line graphs shown in Supplementary Figure S5C and S6C, northern blot signals were quantified at the center of each lane, using FIJI (11). Afterwards, peaks were manually aligned to correct for minor variations in size separation between samples. The average of five neighboring pixels was plotted to smoothen the line graph.
For the quantification of responder piRNA signals in Figures 3C-D, brightness and contrast was adjusted using the window/level function in FIJI to correct for background signal. Subsequently, the piRNA size range (24-33nt) was extrapolated from the EtBr stained marker, responder piRNA signal inside this range was quantified and normalized to the EtBr signal of rRNA to correct for variations in the amount of total RNA that was loaded on the gel.

Generation of small RNA deep sequencing libraries

Small RNA deep sequencing libraries were generated using the NEBNext Small RNA Library Prep Set for Illumina (E7560, New England Biolabs), using 1 µg RNA as input. As piRNAs generally have 2'-*O*-methylated 3' ends, we performed the 3' adapter ligation for 18 hrs at 16°C to enhance the ligation efficiency of small RNAs bearing such modifications. The rest of the library preparation was performed in accordance with the manufacturer's instructions. Libraries were sequenced on an Illumina Hiseq4000 machine by the GenomEast Platform (Strasbourg, France). Sequence data have been deposited in the NCBI sequence read archive under SRA accession SRP272125.

Bioinformatic analyses of small RNA sequences

Quality control and small RNA mapping

RTA 2.7.3 and bcl2fastq were used for image analysis and base calling, and initial quality control was performed using FastQC. All subsequent manipulations were performed in Galaxy (12). First, the FASTX Clip adapter sequences tool (Galaxy version 1.0.3; default settings) was used to clip 3' adapters from the small RNA sequence reads. Subsequently, reads were mapped to the corresponding recombinant Sindbis virus genomes, transposable element sequences extracted from TEFAM (originally downloaded from tefam.biochem.vt.edu on 10-12-2019, now deposited at github.com/RebeccaHalbach/Halbach_tapiR_2020/blob/master/Data/TEfam.fa), *Ae. aegypti* transcripts (AaegL5.2 gene set, downloaded from VectorBase) and the Phasi Charoen like virus (PCLV) genome as sequenced from Aag2 cells (Genbank accession numbers KU936055, KU936056 and KU936057) (13). Reads were mapped using Bowtie allowing 1 mismatch, except for the mapping to interval and desert viruses and for 3' end sharpness, downstream nucleotide bias, and phasing analyses, in which cases no mismatches were

allowed. Small RNA libraries were normalized to the number of reads mapping to published pre-miRNA sequences deposited in miRBase v.21 without allowing mismatches.

Determining piRNA frequencies, size distributions and genome profiles

Size profiles were obtained by counting piRNA lengths after mapping, with the exception of the size profile of small RNAs obtained after PIWI IP (Figure S3), for which raw reads prior to mapping were analyzed from our previously published data (3). For genome distribution plots, the number of piRNA 5' ends was determined for each position of the sequence to which small RNAs have been mapped.

For the analyses of piRNA production from the area of the non-coding reporter RNA downstream of the initiator/responder site versus the remainder of the viral genome shown in Figure 6G and Figure S7E, the SINV genome was defined as the area encompassing the 5' UTR and the non-structural and structural polyproteins up to the start of the duplicated subgenomic promoter (nt 1-11385), and the trailer cassette as nt 11610-11753.

Size distribution and nucleotide biases for individual piRNAs

To generate heat maps of the length profile of piRNAs with a shared 5' end published small RNA sequencing data was re-analyzed (2). SAM files were converted into interval files and piRNA-sized reads (25-30 nt) were filtered and separated according to the strand they mapped to. For each piRNA species, defined by a shared 5' end, the percentage of reads with different lengths relative to the total read count was determined. piRNAs that were supported by less than 20 reads in total were discarded from the analysis. Subsequently, the percentages of read lengths per individual piRNA was imported into Multiple experiment viewer (v 4.9.0; tm4) and k-means clustering based on Pearson correlation was performed using the indicated number of clusters and a maximum number of iterations of 500. The 'Construct hierarchical tree' option was enabled. The graphical outputs of the clustering analyses were combined in Adobe Illustrator.

To analyze nucleotide biases within and downstream of piRNAs, only piRNA sequences that were supported by at least 20 reads and had a dominant piRNA length (at least 75% of all mapped reads have the same length) were considered. Combining the Get flanks (v1.0.0) and Get genomic DNA (v3.0.3) tools, the genomic sequence 5 nt downstream of that dominant piRNA length was extracted. Subsequently, each piRNA species was collapsed to one unique FastA sequence and its corresponding downstream sequence. The sequence logo was generated from these sequences using the Sequence logo generator (Galaxy version 3.5.0) The piRNA logos were cropped in Adobe Illustrator to show nucleotide biases of piRNA positions 1 to 5, 8 to 12, and the downstream positions +1 to +5.

Analysis of general piRNA sharpness

To analyze the effect of Zuc knockdown on vpiRNA 3' ends, small RNAs mapping to the SINV genome up to the subgenomic promotor (nt 1-11385) were considered. For each of the six datasets (3x dsLuc and 3x dsZuc) available for the *gypsy*-25U virus, piRNA species were extracted by selecting only those piRNA start sites that were supported by at least fifty mature (25-30 nt) piRNA reads in the combined six datasets. For these piRNA positions, all reads in the size range of 25-38 nt were extracted from the original mapped data. As for the heat map analysis, the percentage of piRNA lengths was calculated for each piRNA species, defined by a shared 5' end. To reduce noise due to low read count, only the 275 most abundant vpiRNA species were considered, which corresponded to approximately 160,000 reads in the combined six datasets. From the length distribution, a sharpness score was calculated for each piRNA species based on the maximum entropy (S_{max} = all reads have the same length) minus the observed Shannon entropy (S_{obs}) an approach similar to determining nucleotide biases (14): $S_{sharp} = S_{max} - S_{obs} = \log_2(14) - \sum_{n=25}^{38} p_n * \log_2 p_n$

In this formula, the maximum score is defined as log2 of the number of different lengths that a piRNA could possibly have (25 to 38 nt); p_n is defined as the proportion of piRNA reads of a specific length (n) compared to all reads. The mean sharpness score for each piRNA was determined for the three control and three Zuc knockdown datasets separately and the 275

piRNAs were then ranked based on the mean sharpness score in the control knockdown from high score (sharp 3' end) to lower scores (more diffuse 3' end). Within this ranking an average of the mean sharpness scores was then calculated for 11 bins of 25 piRNA species each, both for the control and the Zuc knockdown. Finally, the difference in sharpness score (ΔS_{sharp}) between control and Zuc knockdown was determined for each bin. The entire analysis was repeated for the six datasets of the *gypsy*-28U and *gypsy*-30U viruses and, since the sequence of the three viruses is identical up to the reporter cassette, the data was combined by calculating the mean + SEM of the ΔS_{sharp} per bin obtained for each type of reporter virus. To determine statistical significance, the ΔS_{sharp} was tested against the null hypothesis $\Delta S_{sharp}=0$. A two-sided student's t-test was performed per bin and the hypothesis was rejected at $P < 0.05$. The sharpness score analysis for transposon mapping piRNAs was performed with the 4400 most expressed piRNAs (400 piRNAs per bin).

Analysis of piRNA phasing

piRNA phasing was analyzed for all vpiRNAs from the combined nine dsLuc datasets from the Zuc knockdown experiment (presented in Figure 4). For transposon derived piRNAs this analysis was performed on the combined three dsLuc datasets from the *gypsy-25U* viruses only to ensure that the number of transposon piRNAs reads analyzed was in the same order of magnitude as vpiRNAs. Only piRNAs that mapped to sequences common to all datasets were considered, piRNAs that map to the specific reporter sequences were excluded. First, only piRNA species, defined by a shared 5' end, that were supported by at least 5 reads were selected. For these piRNAs, the distance of every individual piRNA 5' end to up to 200 unique downstream piRNA 5' ends, irrespective of read count, was determined using bedtools Closest bed (ClosestBed, Galaxy Version 2.30.0; settings: input A: piRNA 5' ends of all piRNA reads; input B: piRNA 5' ends of unique piRNA 5' ends; report all ties (-b); only overlaps occurring on the **same** strand; report distance enabled; report upstream distances disabled (-d); report 200 closest hits (-k); ignore features in dataset B that overlap A disabled). The frequency of 5'-5' end distances was counted and plotted for a window of 20-150 nt (scatterplot in Figures 6A-B). The smoothened curves were determined using local regression (Loess; 10% of points fit; Kernel: Epanechnikov) in IBM SPSS v25.

Generation of Zuc expression plasmids

The sequence encoding the Zuc protein was amplified from Aag2 cDNA using CloneAmp HiFi PCR Premix (Takara) and cloned into the pAWG vector (The Drosophila Gateway Vector Collection, Carnegie Science) using In-fusion technology (Takara). The following primers were used to amplify the vector and insert:

Primer name	Nucleotide sequence (5' - 3')
FW-pAWG-INV	GACCCAGCTTTCTTGTACAAAGTGG
RV-pAWG-INV	GGTGTTTCTATCTCCTTCGAAGCC
FW-Zuc_C	GGAGATAGAAACACCATGATGTTGGAATCTGTGCCACG
RV-Zuc_C	CAAGAAAGCTGGGTCCTGCCTATCTCCCCCGATGG

Subsequently, pAW3F-Zuc was generated by inverse PCR of pAWG-Zuc, which introduced the 3×flag tag to replace the eGFP-tag, followed by In-fusion cloning (Takara Biotech). The primers used for this inverse PCR are shown below (underlined sequence makes up the 3×flag tag, double underlined sequence is the 15 nt overlap required for In-fusion cloning):

Primer name	Nucleotide sequence (5' - 3')
FW_pAW3F-Zuc	ATAAGGACGACGATGATAAGGACTACAAGGACGACGACGATAAGCACCGGTCCACGTGACG
RV_pAW3F-Zuc	CATCGTCGTCCTTATAATCCTTGTCATCGTCGTCTTTGTAATCGGTGGCGGAGCTCACC

Immunofluorescence

Approximately 5×10^5 Aag2 cells were transfected with 1 µg of the pAW3F-Zuc expression vector using 1 µL X-tremeGENE HP DNA Transfection Reagent (Roche) according to the manufacturer's instructions. 48 hrs later, cells were fixed on coverslips using 4% paraformaldehyde for 10 minutes at room temperature. Fixed cells were permeabilized in PBS-Triton (0.25%) for 10 minutes and blocked in 10% normal goat serum/0.3M Glycine in

PBS-Tween (0.1%). Cells were stained with mouse anti-flag antibody (1:200, Sigma, F1804, RRID: AB_262044) for 1 hr at room temperature, followed by goat anti-mouse IgG Alexa fluor 568 (1:200, Invitrogen, A-11004, RRID: AB_2534072) for 1 hr at room temperature. Following antibody staining, mitochondria were stained using 200 µM Mitoview Green (Biotium), according to the manufacturer's instruction. Lastly, Hoechst reagent was used to stain the nuclei and the coverslips were mounted onto microscope slides for imaging using the Zeiss LSM900 confocal microscope. In between all steps during the staining procedure, cells were washed three times with PBS.

Co-immunoprecipitation and western blot

For immunoprecipitation of Zuc, ~4.5 × 10^6 Aag2 cells were seeded and after ~16 hrs, 15 µg of the pAW3F-Zuc was transfected using X-tremeGENE HP DNA Transfection Reagent (Roche) according to the manufacturer's instructions. Cells were lysed 48 hrs after transfection using 300 µL lysis buffer (10 mM Tris-HCl pH 7.5, 150 mM NaCl, 0.5 mM EDTA, 0.5% Igepal CA-630 [Sigma], 10% Glycerol, 1x cOmplete Protease Inhibitor [Roche], 1 mM PMSF). After incubation for 1 hr at 4°C with end-over-end rotation, lysates were centrifuged for 30 min at 15000 × g, 4°C. The supernatant was snap-frozen in liquid nitrogen and stored at -80°C for later use.

For immunoprecipitation, 15 µL M2-Flag bead slurry (Sigma) was equilibrated in lysis buffer, and, along with 450 µL dilution buffer (10 mM Tris-HCl pH 7.5, 150 mM NaCl, 0.5 mM EDTA, 1x cOmplete Protease Inhibitor [Roche], 1 mM PMSF), added to the thawed lysate. After incubation for 2 hrs at 4°C with end-over-end rotation, beads were washed thrice using 500 µL dilution buffer, before harvesting immunoprecipitates by boiling for 10 min in 20 µL 2× sample buffer (120 mM Tris/Cl pH 6.8, 20% glycerol, 4% SDS, 0.04% bromophenol blue, 10% β-mercaptoethanol). After boiling, samples were diluted by adding 20 µL lysis buffer.

Samples were resolved on 10% polyacrylamide gels and blotted to nitrocellulose membranes. The following antibodies, generated in our laboratory, were used for western blotting: rabbit-anti-Ago3, -Piwi4, -Piwi5 and -Piwi6 (all at 1:500) (3,4). Additionally, mouse anti-flag (1:1000, Sigma, F1804, RRID: AB_262044) was used. Secondary antibodies were goat-anti-rabbit-IRdye800 [Li-cor; 926-32211, RRID: AB_621843] and goat-anti-mouse-IRdye680 [926-68070, RRID: AB_10956588].

Supplemental References

1. Palatini, U., Miesen, P., Carballar-Lejarazu, R., Ometto, L., Rizzo, E., Tu, Z., van Rij, R.P. and Bonizzoni, M. (2017) Comparative genomics shows that viral integrations are abundant and express piRNAs in the arboviral vectors Aedes aegypti and Aedes albopictus. *BMC Genomics*, **18**, 512.
2. Miesen, P., Girardi, E. and van Rij, R.P. (2015) Distinct sets of PIWI proteins produce arbovirus and transposon-derived piRNAs in Aedes aegypti mosquito cells. *Nucleic Acids Res*, **43**, 6545-6556.
3. Joosten, J., Miesen, P., Taskopru, E., Pennings, B., Jansen, P., Huynen, M.A., Vermeulen, M. and Van Rij, R.P. (2019) The Tudor protein Veneno assembles the ping-pong amplification complex that produces viral piRNAs in Aedes mosquitoes. *Nucleic Acids Res*, **47**, 2546-2559.
4. Halbach, R., Miesen, P., Joosten, J., Taskopru, E., Rondeel, I., Pennings, B., Vogels, C.B.F., Merkling, S.H., Koenraadt, C.J., Lambrechts, L. *et al.* (2020) A satellite repeat-derived piRNA controls embryonic development of Aedes. *Nature*, **580**, 274-277.
5. Hahn, C.S., Hahn, Y.S., Braciale, T.J. and Rice, C.M. (1992) Infectious Sindbis virus transient expression vectors for studying antigen processing and presentation. *Proc Natl Acad Sci U S A*, **89**, 2679-2683.
6. Saleh, M.C., Tassetto, M., van Rij, R.P., Goic, B., Gausson, V., Berry, B., Jacquier, C., Antoniewski, C. and Andino, R. (2009) Antiviral immunity in Drosophila requires systemic RNA interference spread. *Nature*, **458**, 346-350.
7. Vodovar, N., Bronkhorst, A.W., van Cleef, K.W., Miesen, P., Blanc, H., van Rij, R.P. and Saleh, M.C. (2012) Arbovirus-derived piRNAs exhibit a ping-pong signature in mosquito cells. *PLoS One*, **7**, e30861.
8. Livingstone, C.D. and Barton, G.J. (1993) Protein sequence alignments: a strategy for the hierarchical analysis of residue conservation. *Comput Appl Biosci*, **9**, 745-756.
9. Livak, K.J. and Schmittgen, T.D. (2001) Analysis of relative gene expression data using real-time quantitative PCR and the 2(-Delta Delta C(T)) Method. *Methods*, **25**, 402-408.
10. Pall, G.S. and Hamilton, A.J. (2008) Improved northern blot method for enhanced detection of small RNA. *Nat Protoc*, **3**, 1077-1084.
11. Rueden, C.T., Schindelin, J., Hiner, M.C., DeZonia, B.E., Walter, A.E., Arena, E.T. and Eliceiri, K.W. (2017) ImageJ2: ImageJ for the next generation of scientific image data. *BMC Bioinformatics*, **18**, 529.
12. Blankenberg, D., Gordon, A., Von Kuster, G., Coraor, N., Taylor, J., Nekrutenko, A. and Galaxy, T. (2010) Manipulation of FASTQ data with Galaxy. *Bioinformatics*, **26**, 1783-1785.
13. Maringer, K., Yousuf, A., Heesom, K.J., Fan, J., Lee, D., Fernandez-Sesma, A., Bessant, C., Matthews, D.A. and Davidson, A.D. (2017) Proteomics informed by transcriptomics for characterising active transposable elements and genome annotation in Aedes aegypti. *Bmc Genomics*, **18**, 101.
14. Crooks, G.E., Hon, G., Chandonia, J.M. and Brenner, S.E. (2004) WebLogo: a sequence logo generator. *Genome Res*, **14**, 1188-1190.
